# Supplementary material for: In Vitro Antiplasmodial and Cytotoxic Activities of Compounds from the Roots of Eriosema montanum Baker f. (Fabaceae)
Source: Molecules. 2021 May 10;26(9):2795. doi: 10.3390/molecules26092795 (PMC8125995; doi:10.3390/molecules26092795)
Supplement: Supplementary file 1 [file molecules-26-02795-s001.zip › molecules-1197824-SI.pdf]

In vitro antiplasmodial and cytotoxic activities of compounds from the roots of *Eriosema montanum Baker f.* (Fabaceae)

Jean Claude Didelot Tomani[1,2], Olivier Bonnet[3], Alain Nyirimigabo[1,3], William Deschamps, Alembert Tiabou Tchinda[4], Olivia Jansen[3], Allison Ledoux[3], Mukazayire Marie Jeanne[1], Luc Vanhamme[2], Michel Frederich[3†], Raymond Muganga[1†] and Jacob Souopgui[2*†]

[1] *School of Medicine and Pharmacy, College of Medicine and Health Sciences, University of Rwanda, PoBox 3286 Kigali, Rwanda*;
[2] *Department of Molecular Biology, Institute for Molecular Biology and Medicine, Université Libre de Bruxelles, 6041 Gosselies, Belgium*;
[3] *Laboratory of Pharmacognosy, Centre for Interdisciplinary Research on Medicines (CIRM), University of Liège*, B36, 4000 *Liège, Belgium;*
[4] *Laboratory of Phytochemistry, Centre for Research on Medicinal Plants and Traditional Medicine, Institute of Medical Research and Medicinal Plants Studies, PO Box 6163 Yaoundé, Cameroon;*
***** Correspondence: jsouopgu@ulb.ac.be; Tel.: +32-2-650-9936
† Shared last authorship.

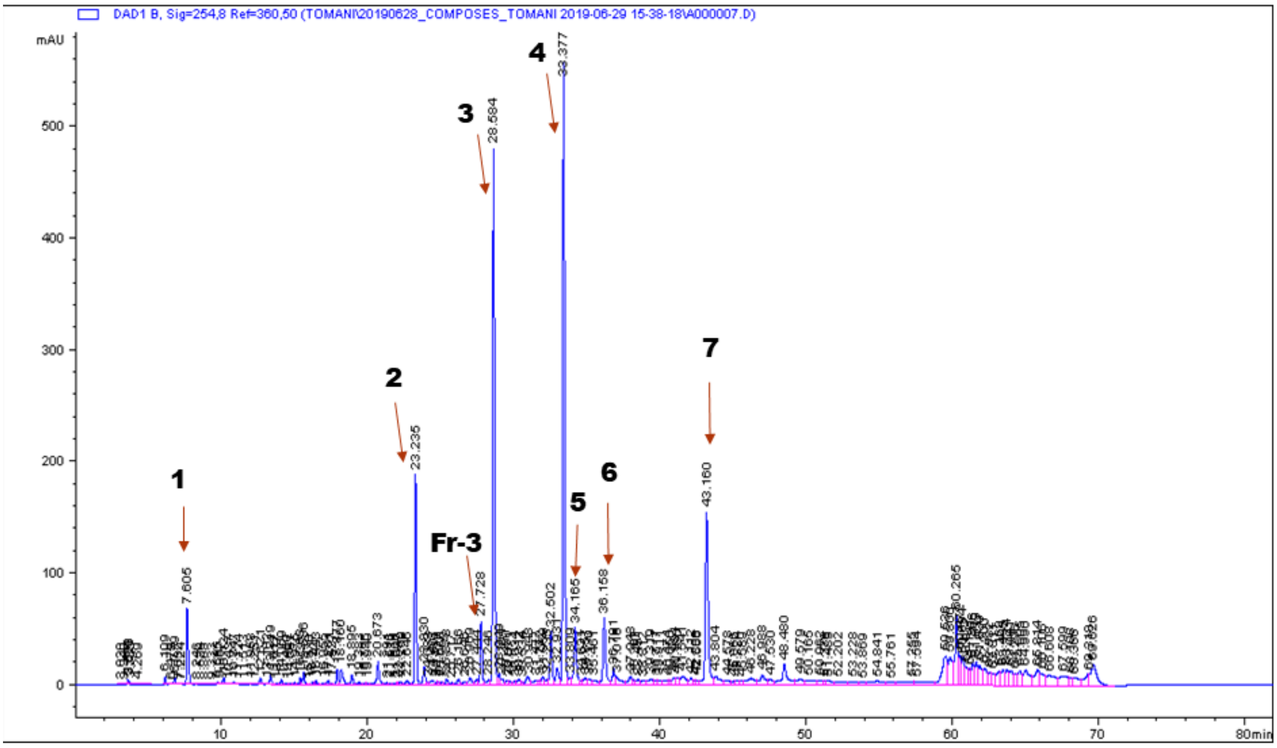

Figure S.1: ***High performance Liquid Chromatography (HPLC) Chromatogram of the crude extract of E. montanum***. An in-house HPLC method used to screen the major components of crude extract was used. Analytical separation was carried out on a Hypersil ODS® RP18 column (250 × 4.6 µm; particle size 5 µm). All samples were dissolved in methanol HPLC-grade, filtered through a 0.45 µm pore size filter membrane and analyzed on an Agilent 1100 HPLC machine. Samples were eluted with a nonlinear gradient method with acetonitrile and 0.05% trifluoroacetic acid in ultra-pure water. The column temperature was maintained at 25 °C. Then 20 µL of each sample were injected into the HPLC-UV/DAD (Diode-Array Detection) system and the analysis, performed at a flow rate of 1.0 mL/min, was monitored at 210, 254, 288, and 350 nm.

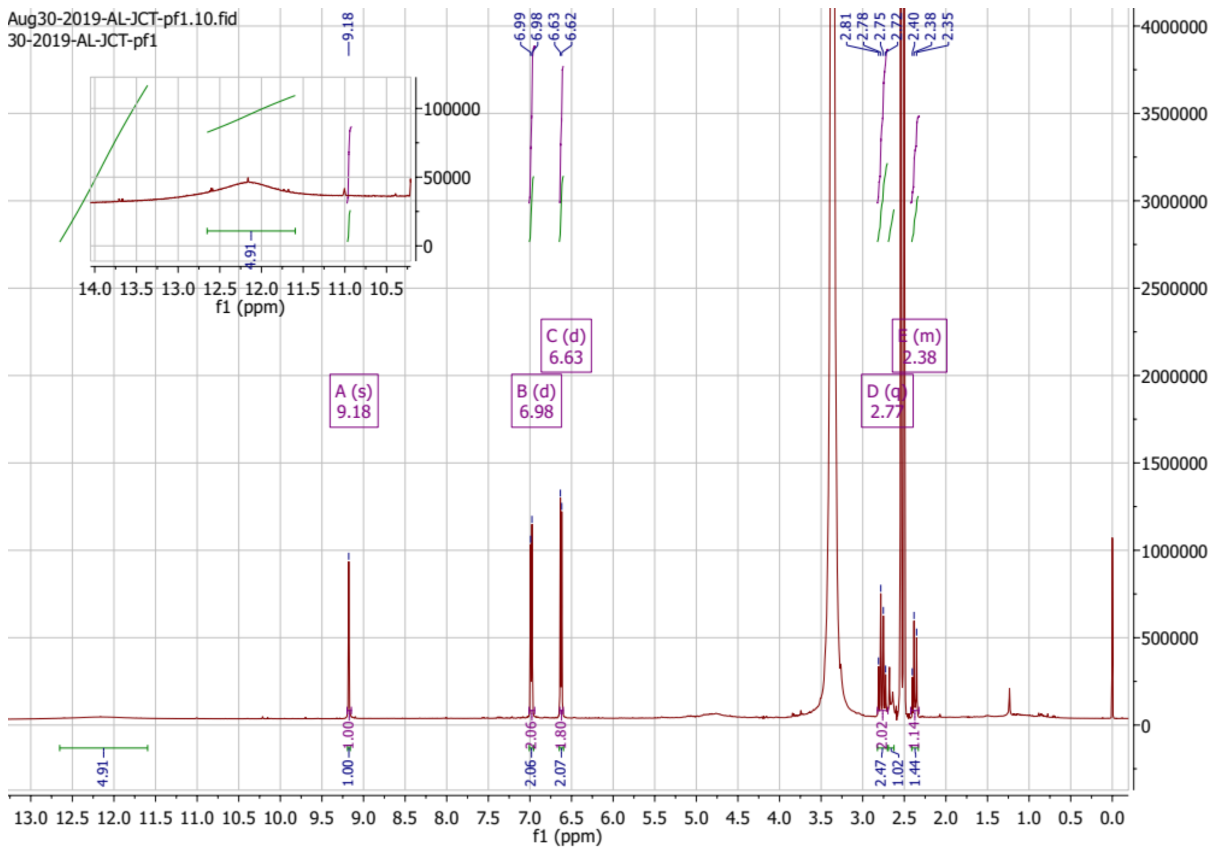

Figure S.2: 1H NMR (500Mhz, DMSO-D6) spectrum of compound 1

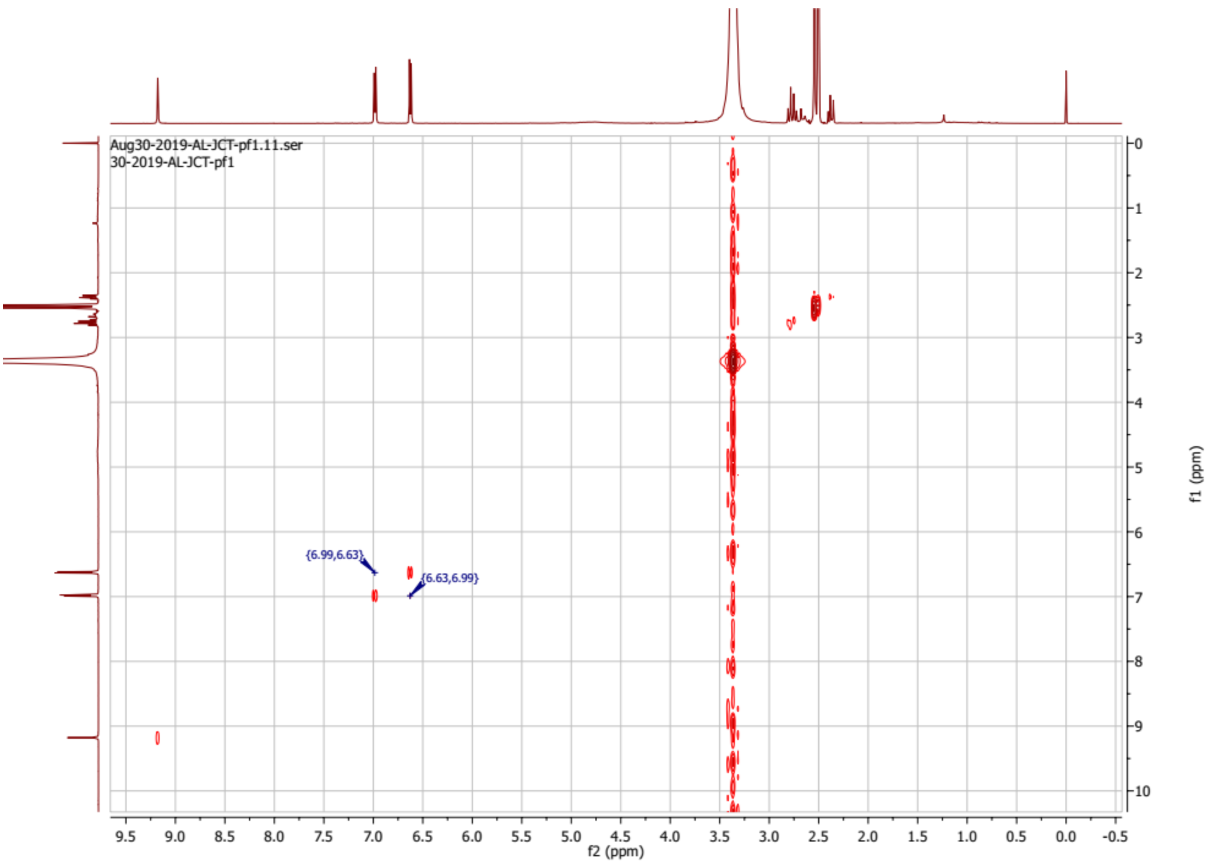

Figure S.3: Cosy NMR (500Mhz, DMSO-D6) spectrum of compound 1

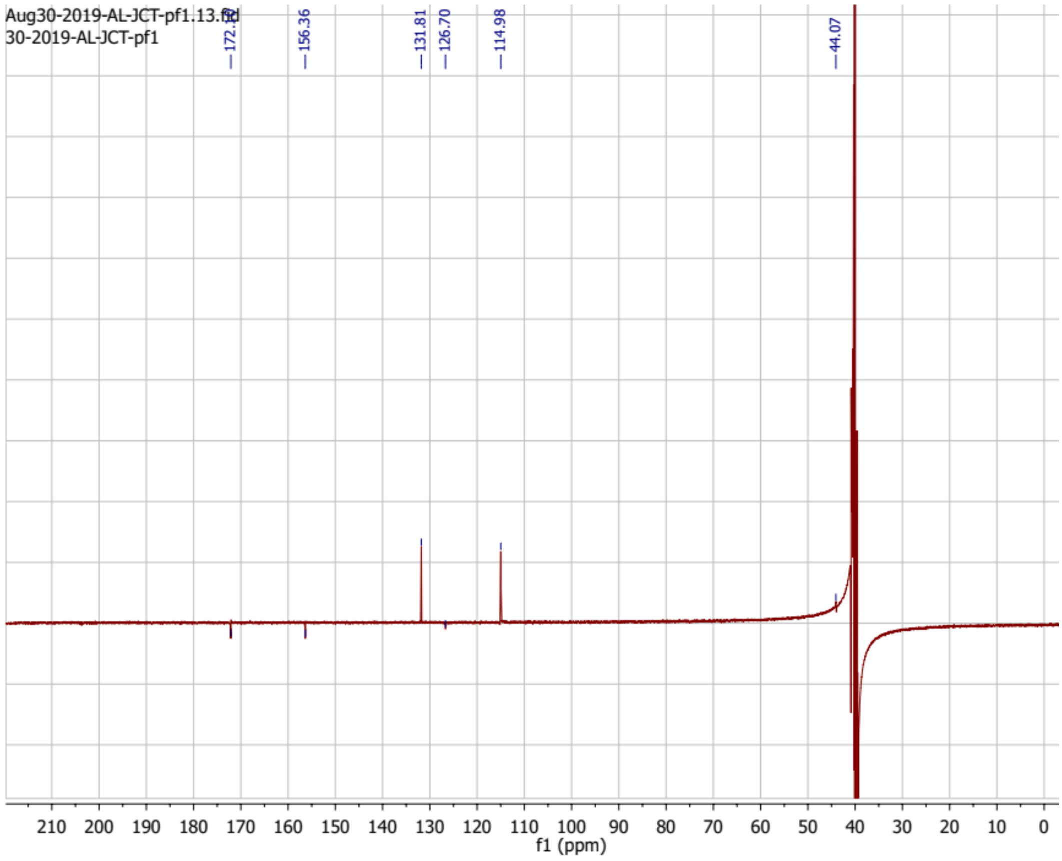

Figure S.4: 13C nmr NMR (500Mhz, DMSO-D6) spectrum of compound 1

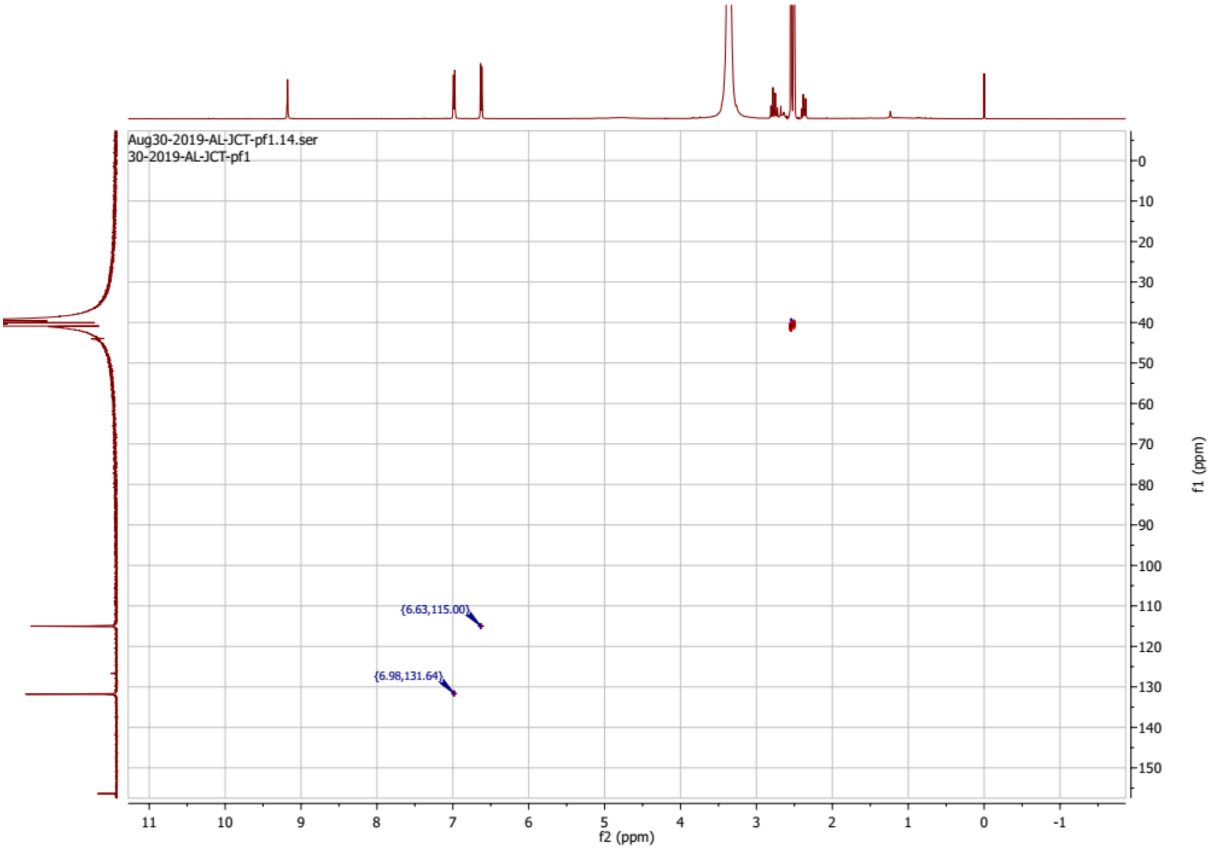

Figure S.5: HSQC (500Mhz, DMSO-D6) spectrum of compound 1

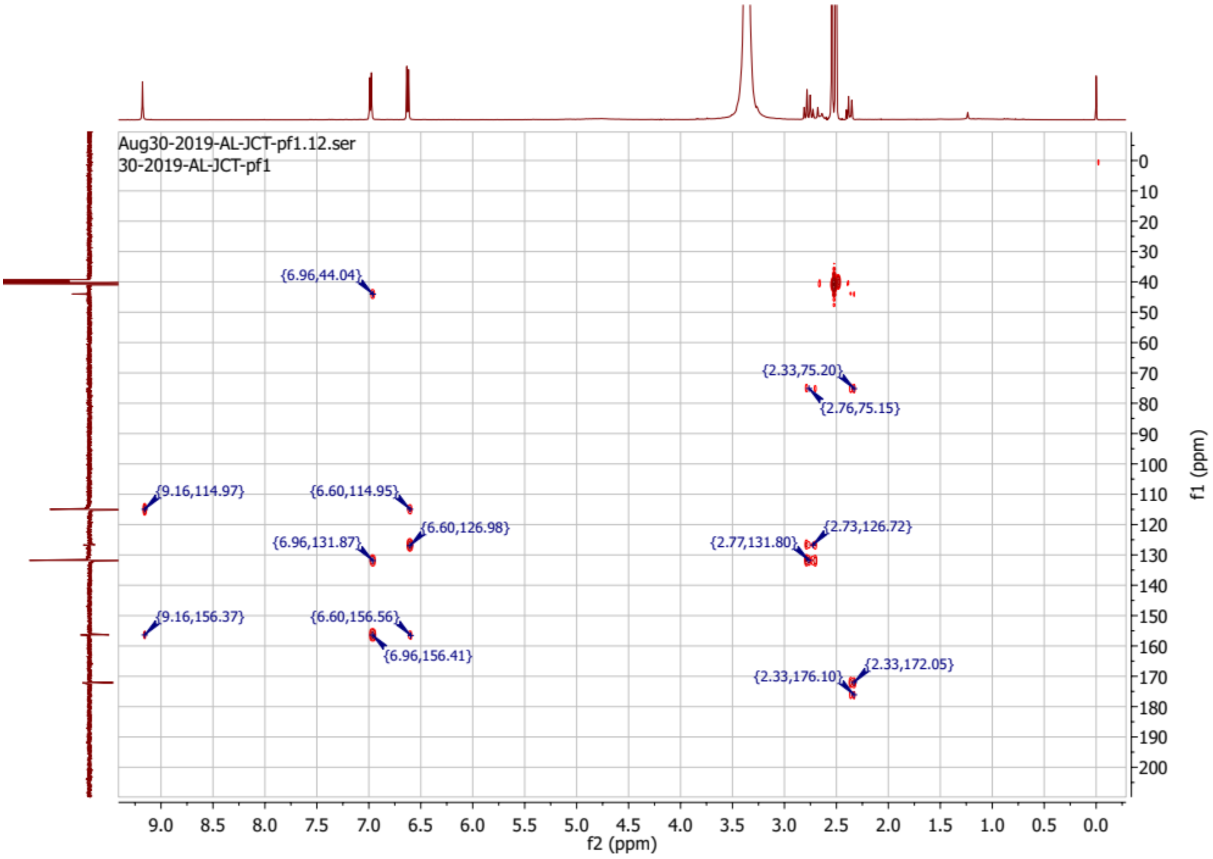

Figure S.6: HMBC (500Mhz, DMSO-D6) spectrum of compound 1

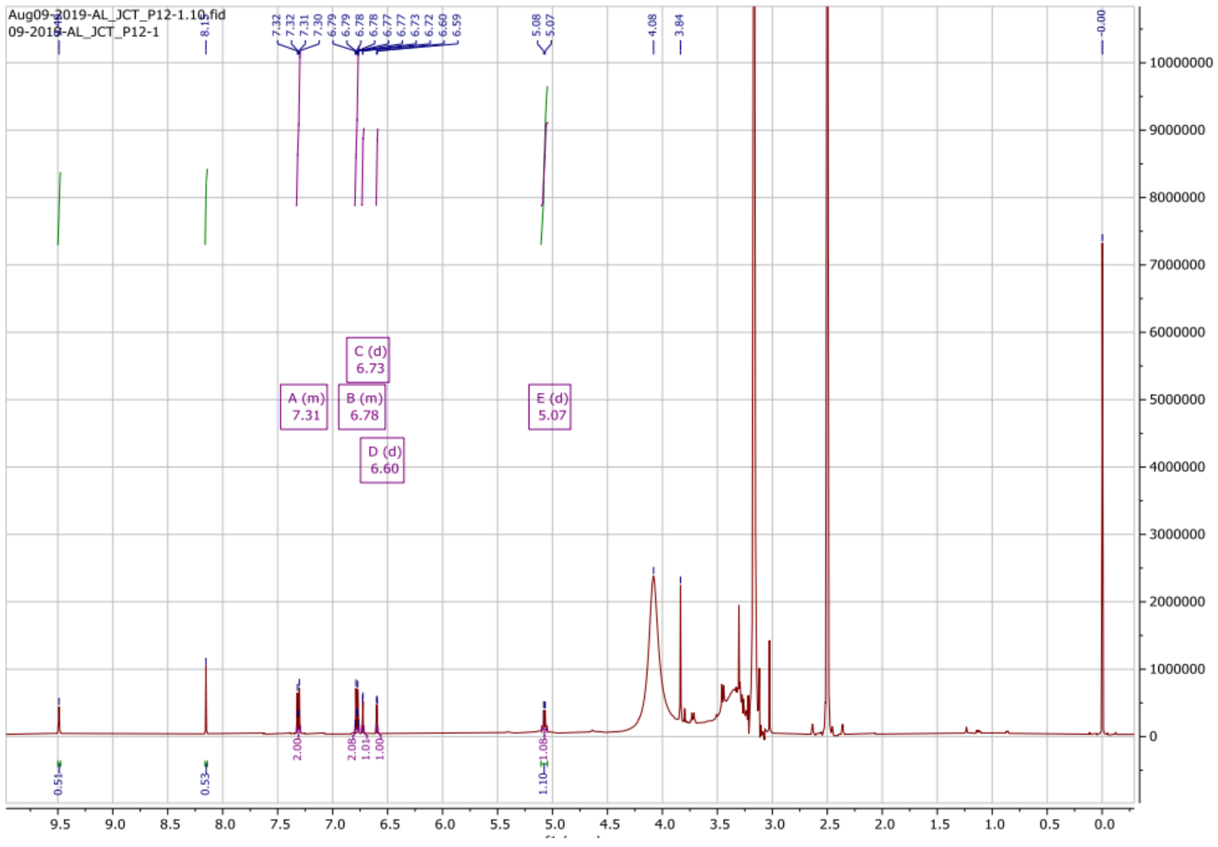

*Figure S.7: 1H NMR (500Mhz, DMSO-D6) spectrum of compound **2***

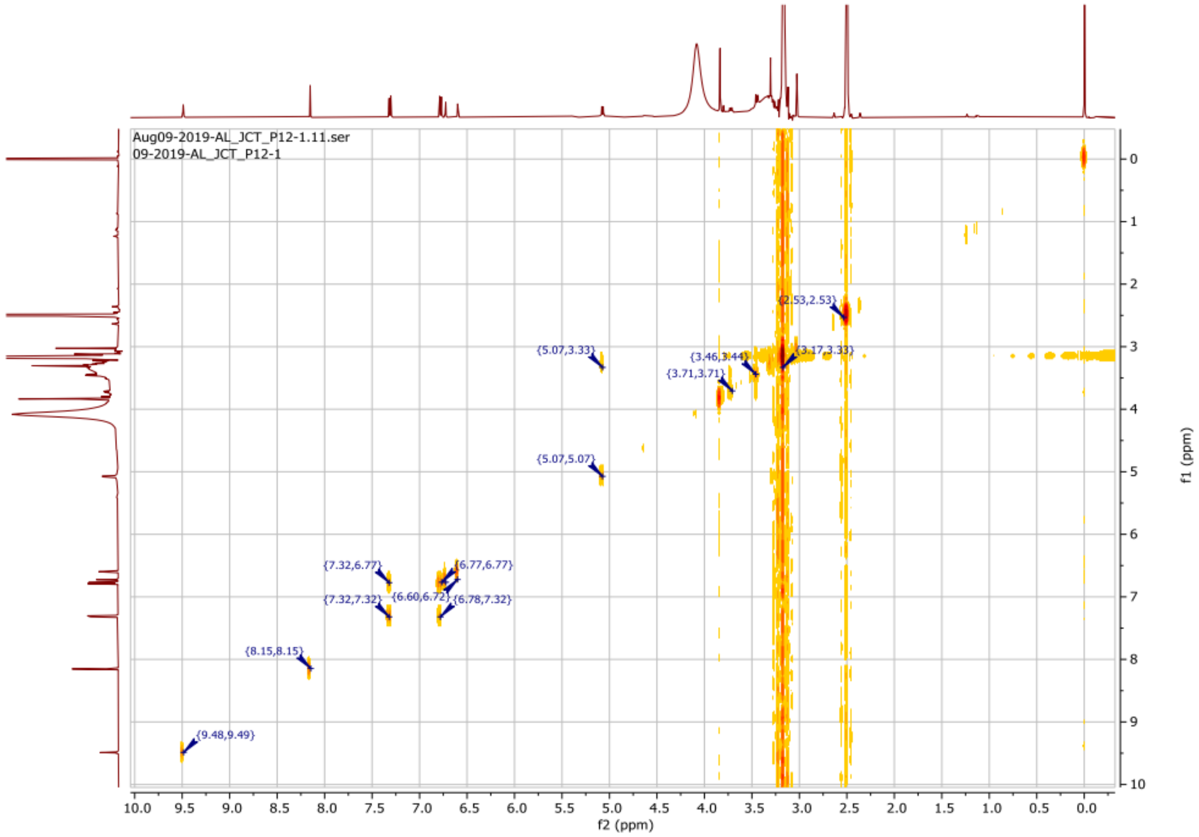

*Figure S.8: Cosy (500Mhz, DMSO-D6) spectrum of compound **2***

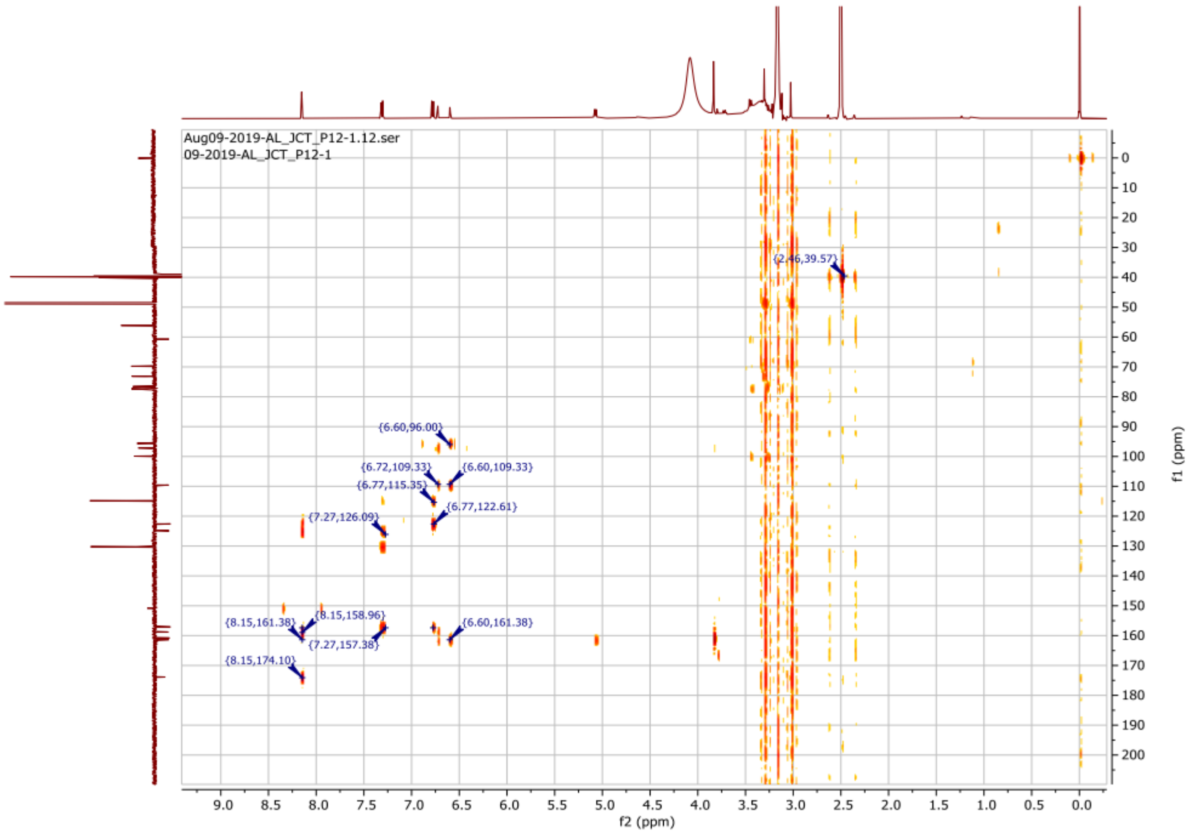

*Figure S.9: HMBC spectrum NMR (500Mhz, DMSO-D6) of the compound **2***

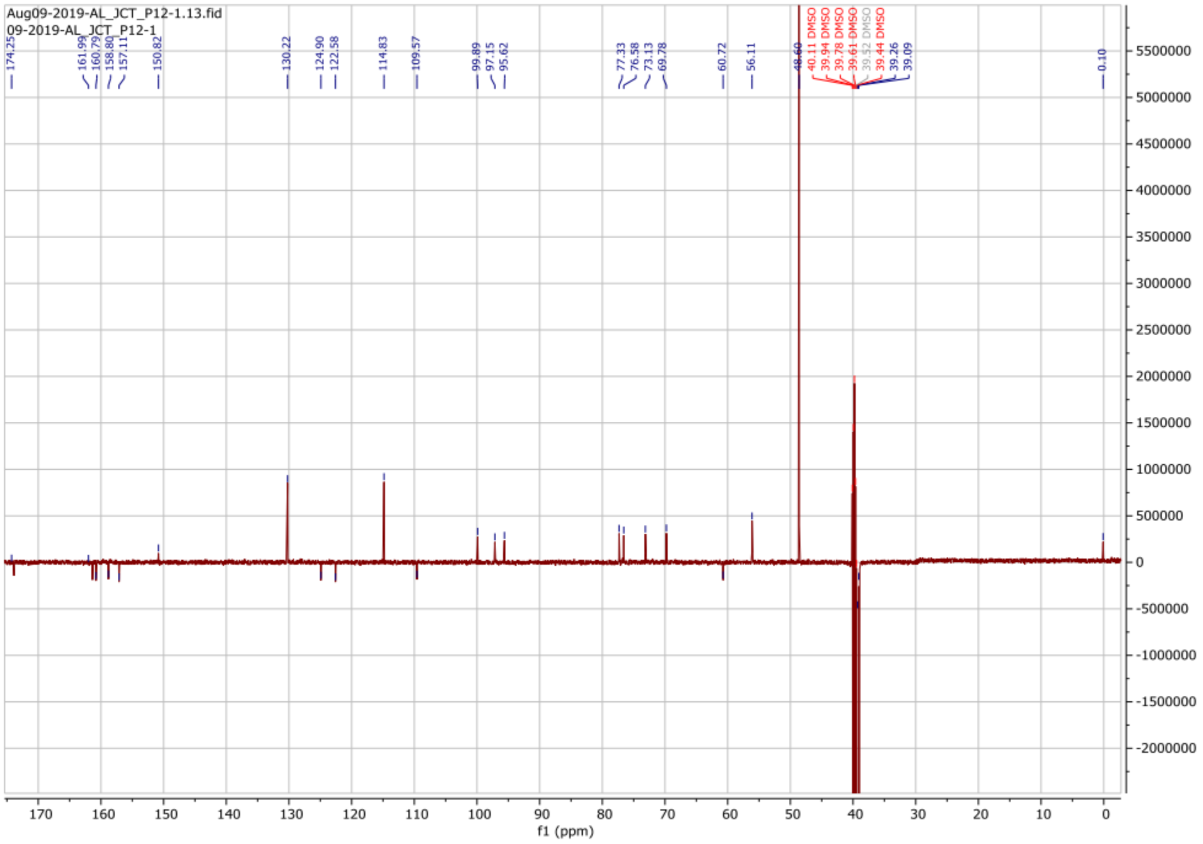

Figure S.10: 13C NMR (500Mhz, DMSO-D6) Spectrum of compound 2

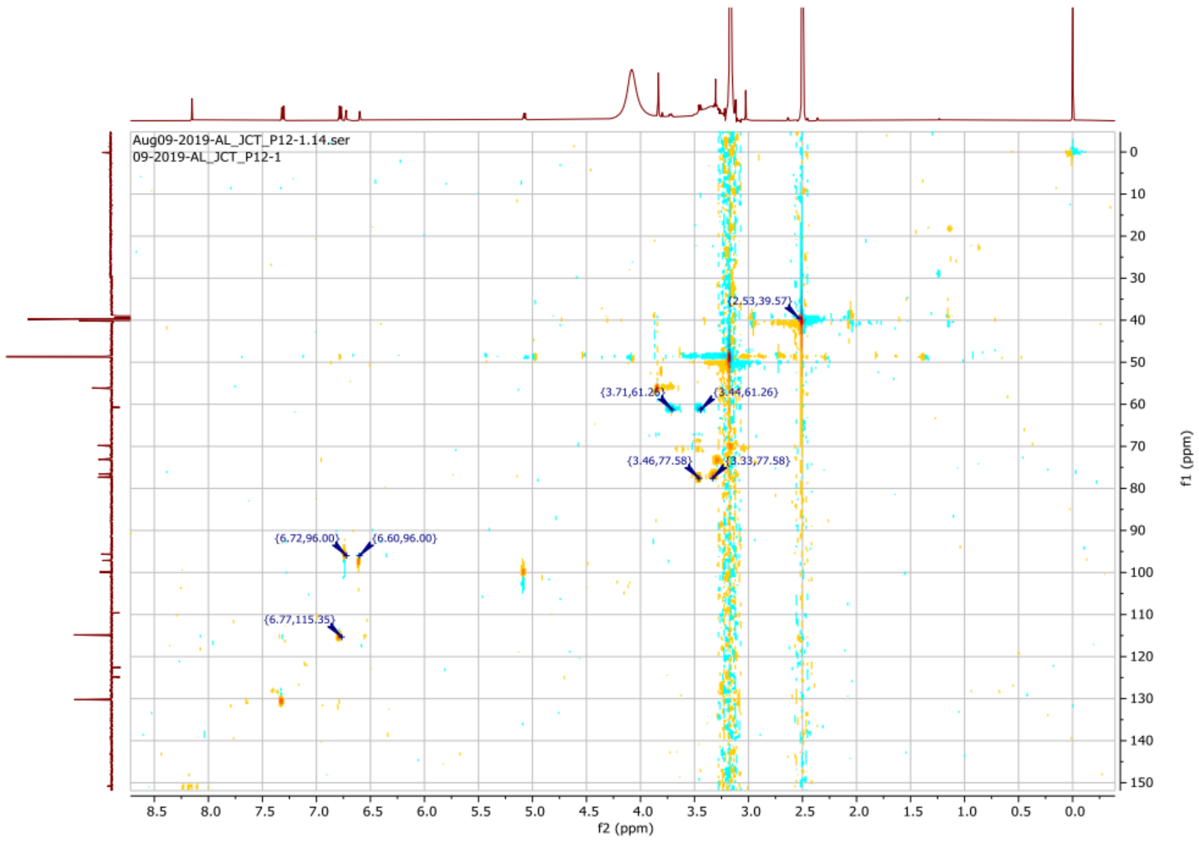

Figure S.11: HSQC spectrum (500Mhz, DMSO-D6) of the compound 2

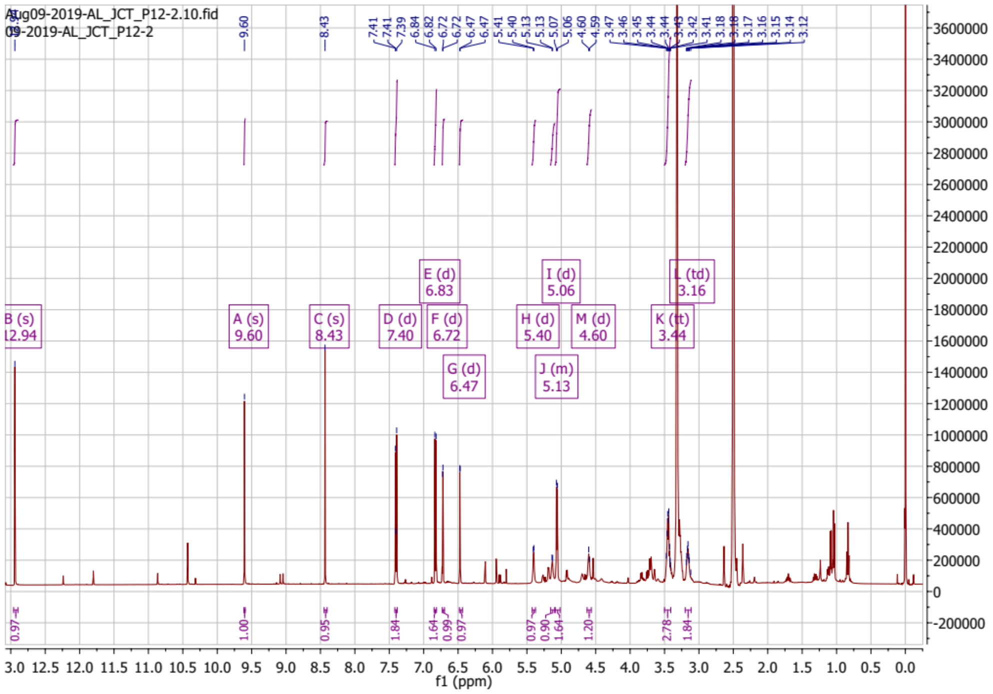

Figure S.12: 1H NMR (500Mhz, DMSO-D6) spectrum for compound 3

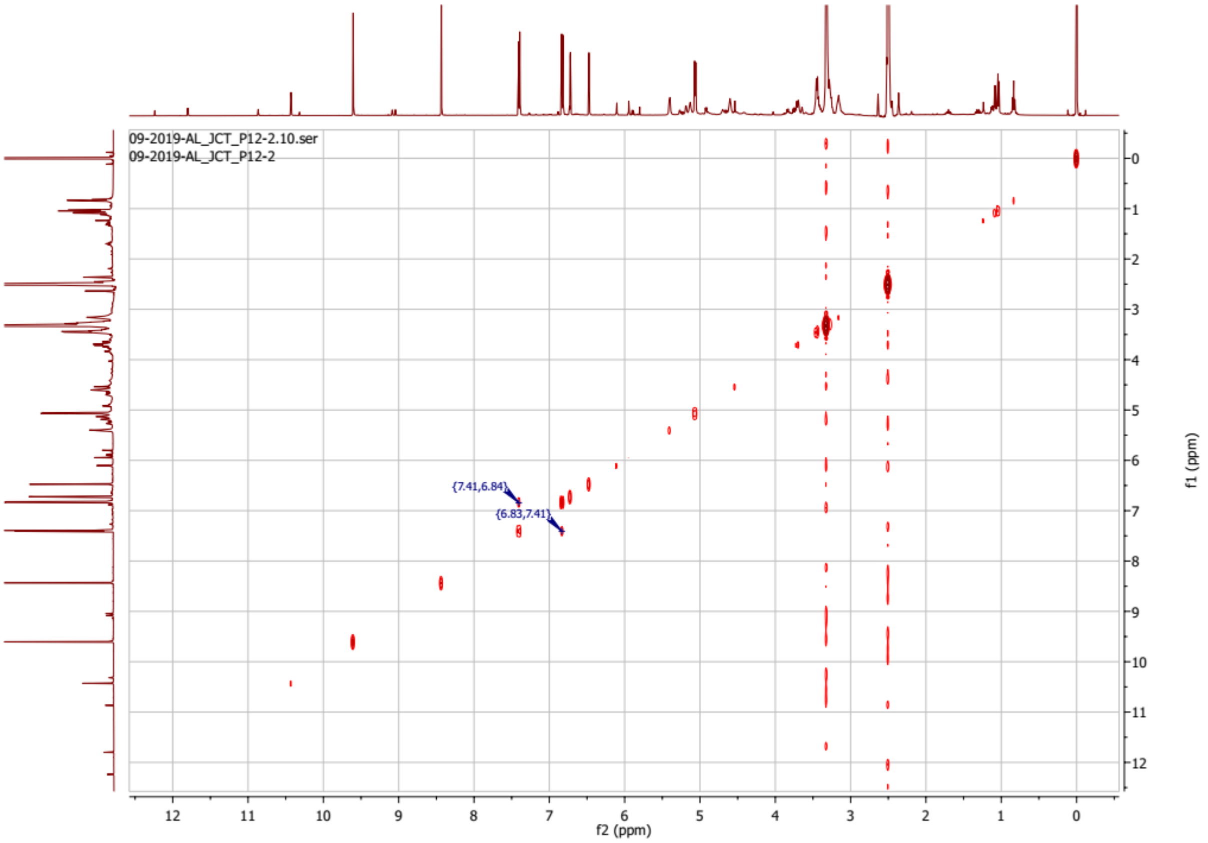

Figure S.13: Cosy spectrum (500Mhz, DMSO-D6) for compound 3

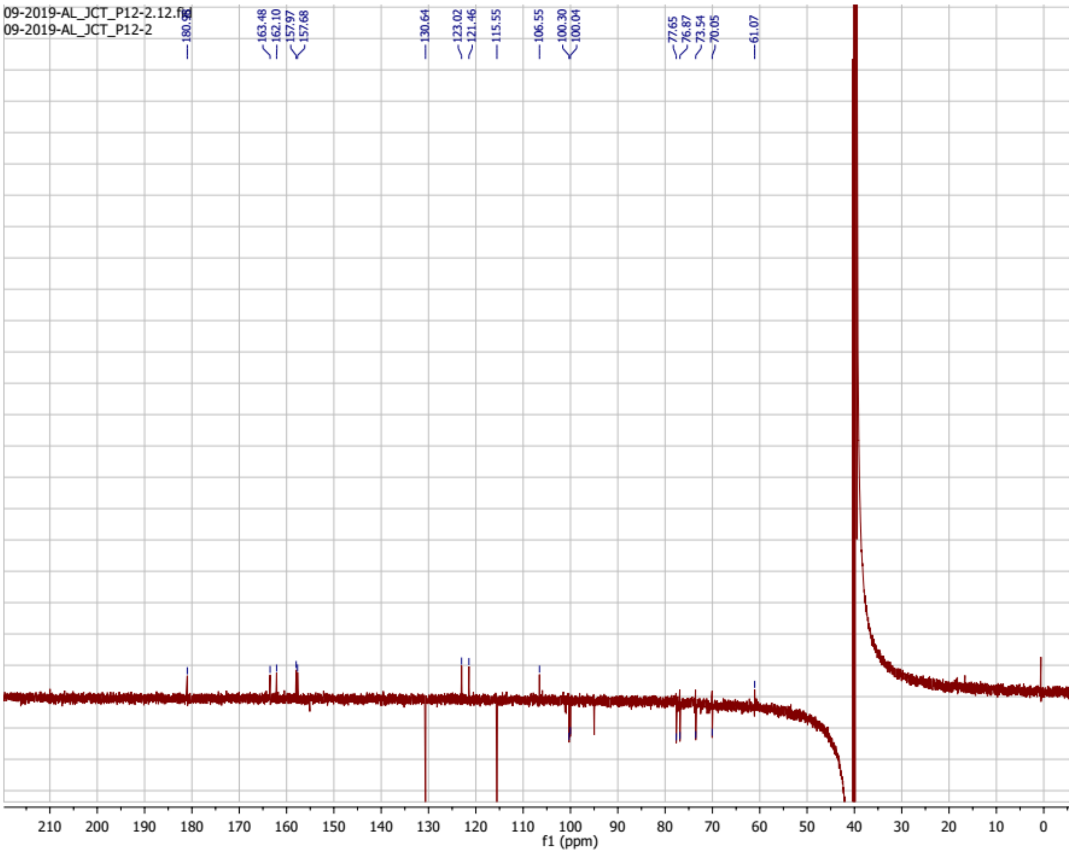

Figure S.14:13C NMR (500Mhz, DMSO-D6) spectrum for compound 3

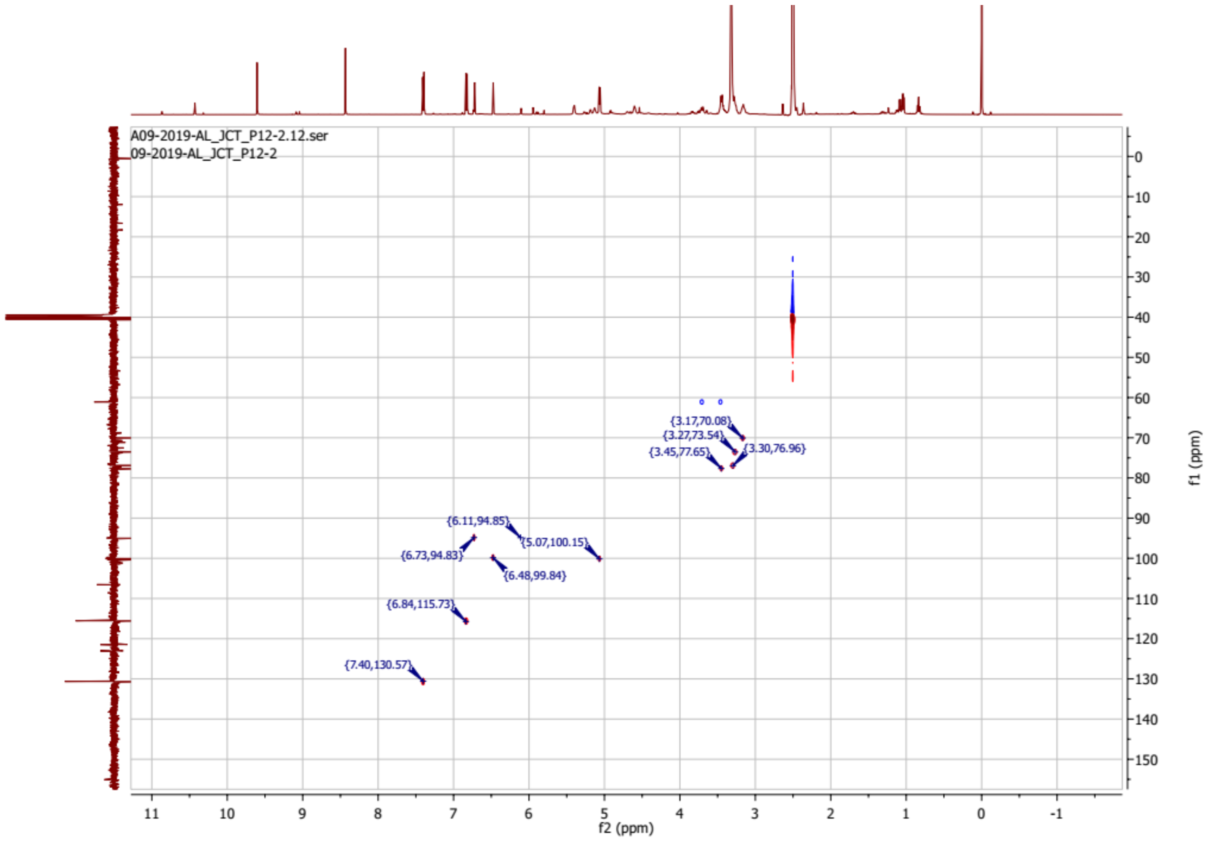

Figure S.15: HSQC spectrum (500Mhz, DMSO-D6) for compound 3

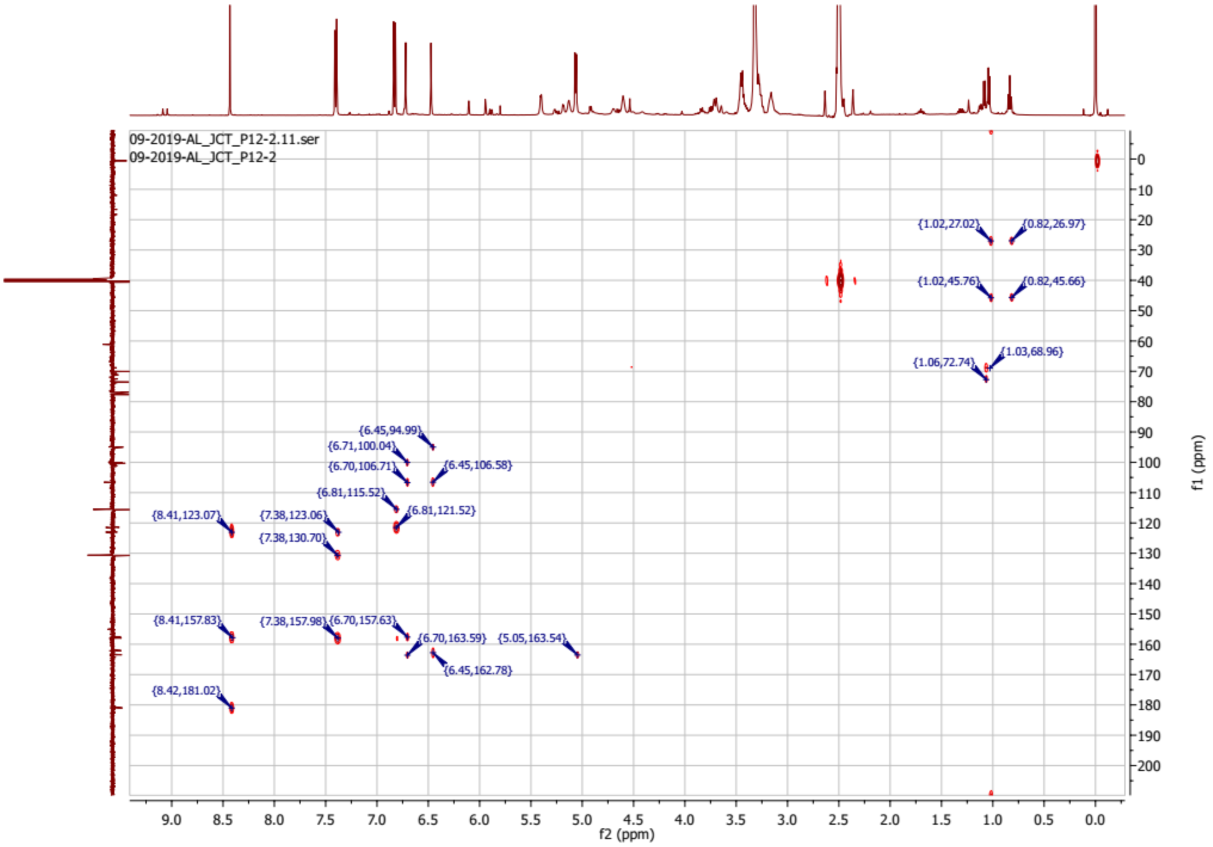

Figure S.16: HMBC spectrum (500Mhz, DMSO-D6) for compound 3

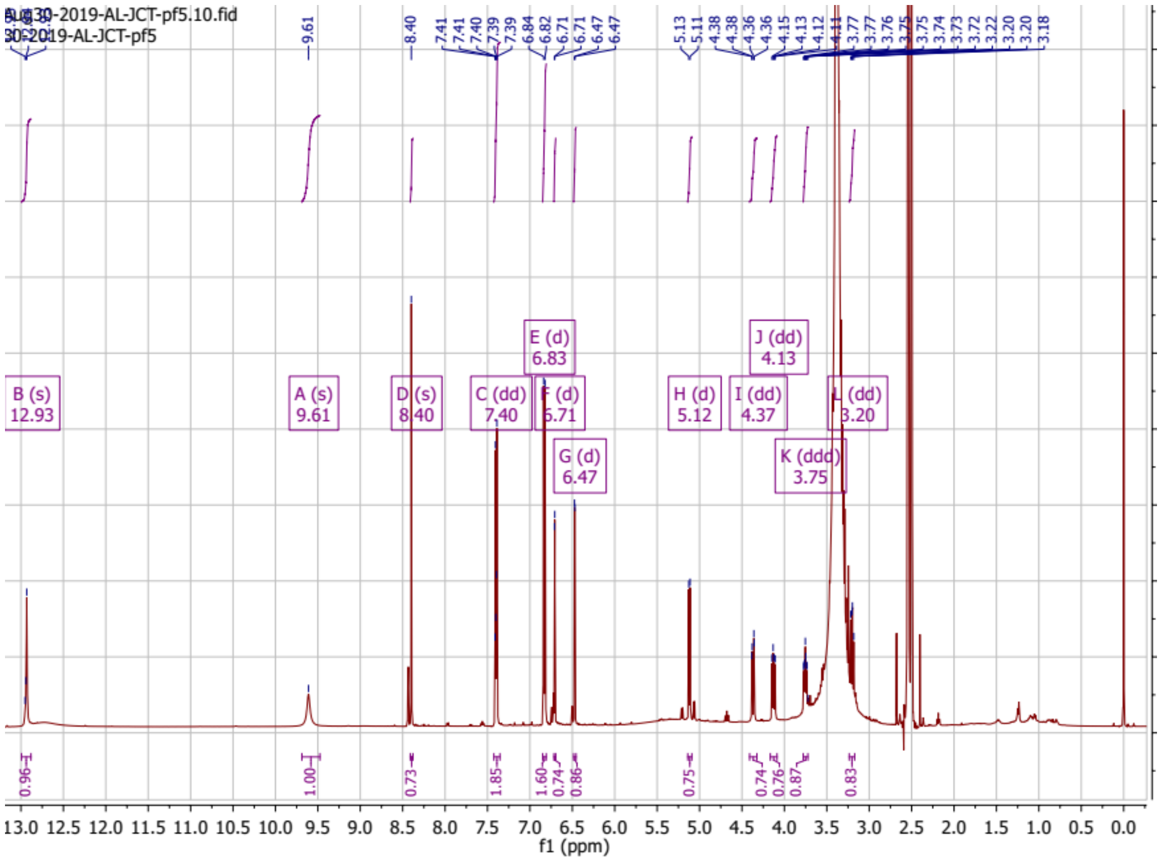

Figure S.17: 1H NMR (500Mhz, DMSO-D6) spectrum of compound 4

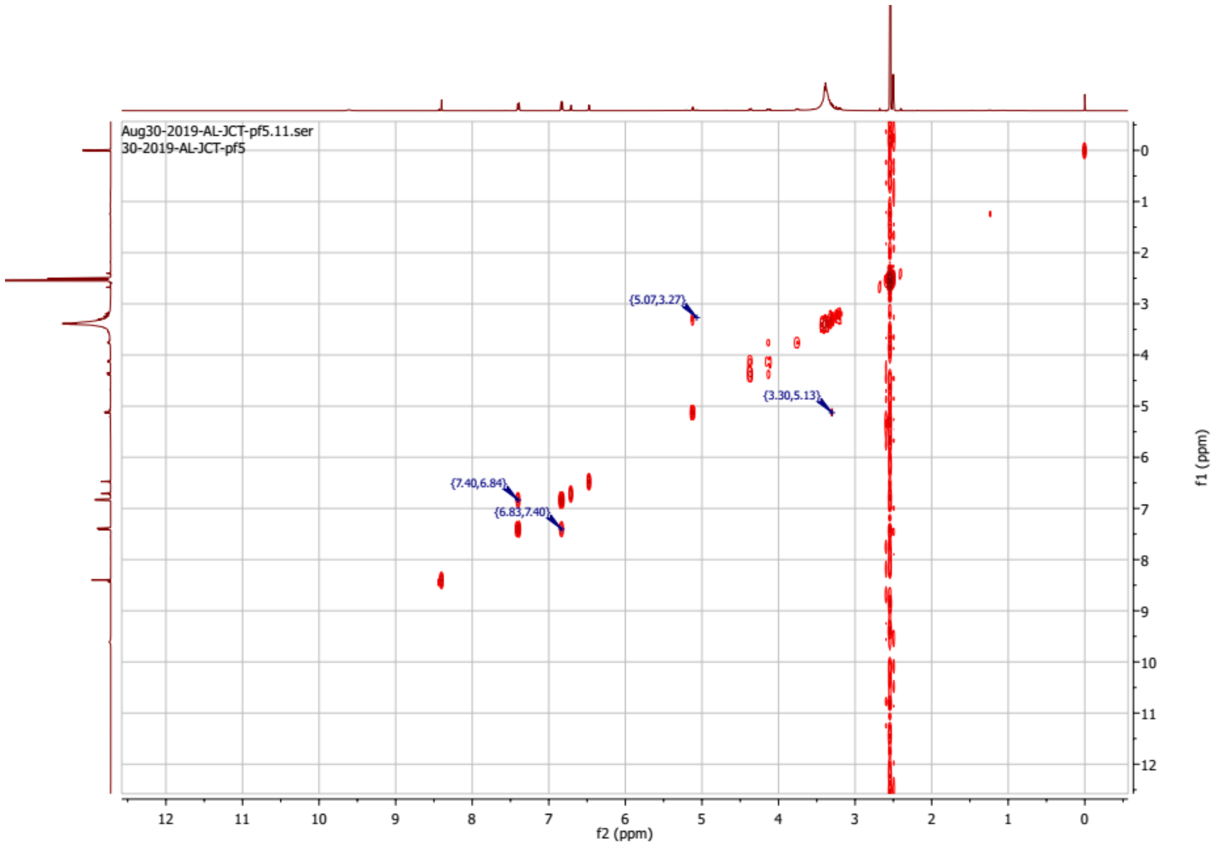

Figure S.18: Cosy spectrum (500Mhz, DMSO-D6) of compound 4

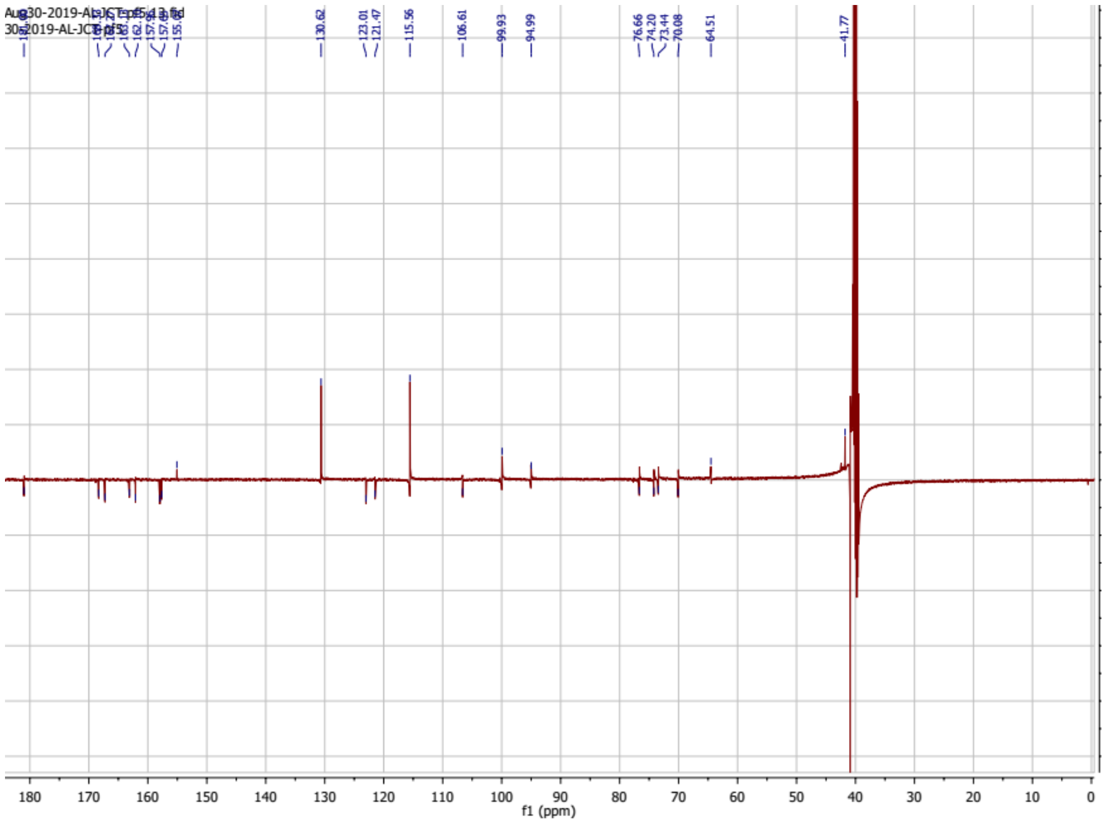

Figure S.19: 13C NMR (500Mhz, DMSO-D6) spectrum for compound 4

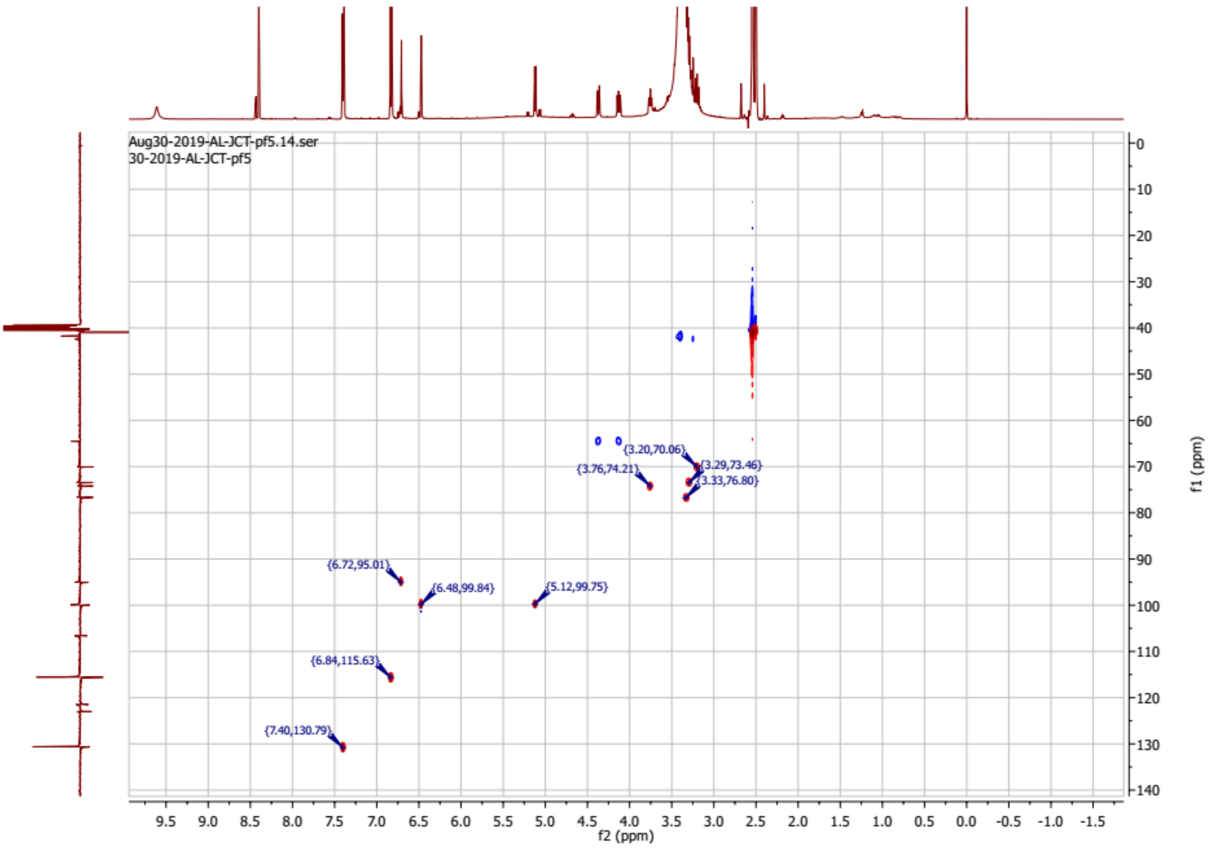

Figure S.20:HSQC spectrum (500Mhz, DMSO-D6) for commpound 4

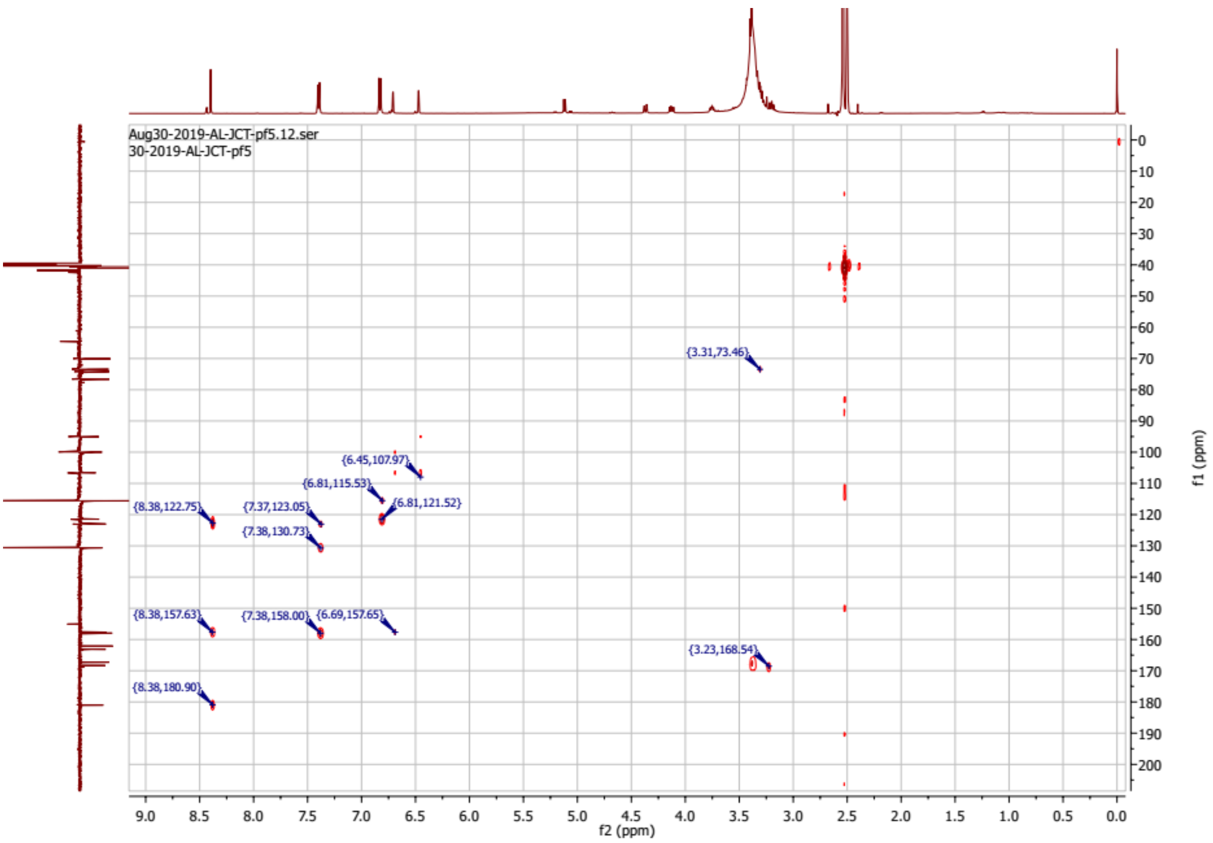

Figure S.21: HMBC spectrum (500Mhz, DMSO-D6) for compound 4

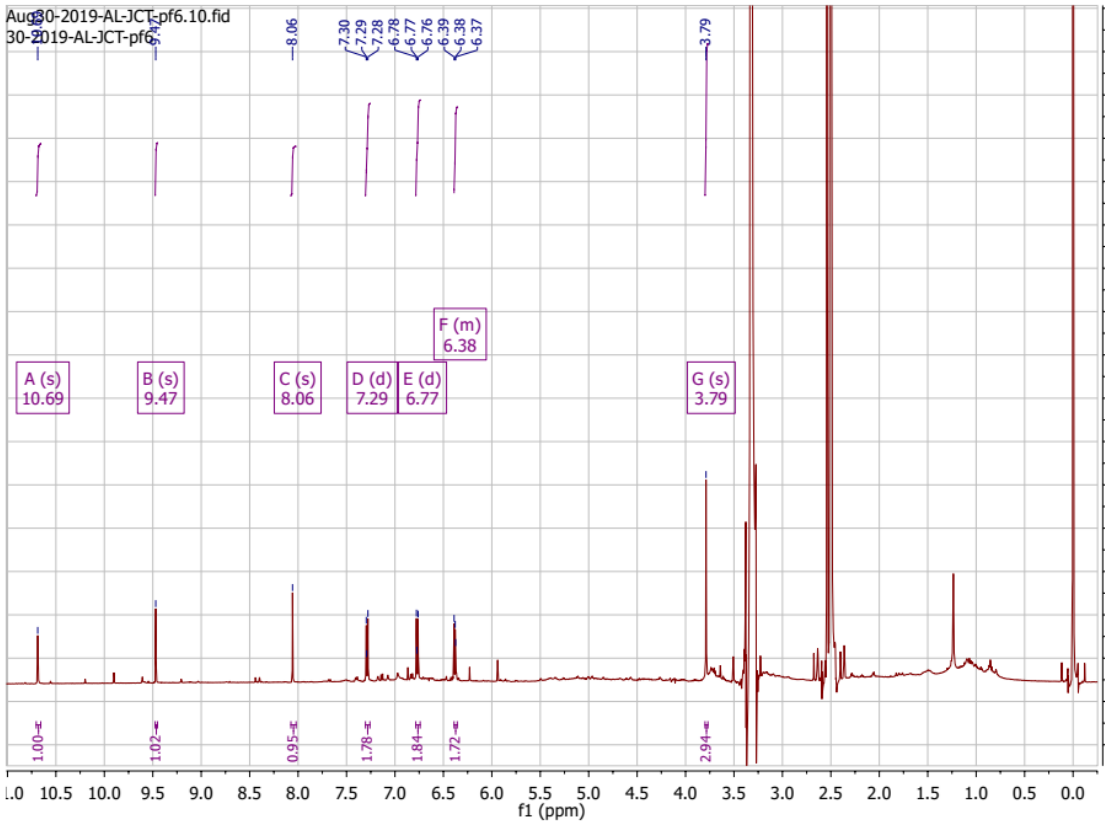

Figure S.22: 1H NMR (500Mhz, DMSO-D6) spectrum for compound 5

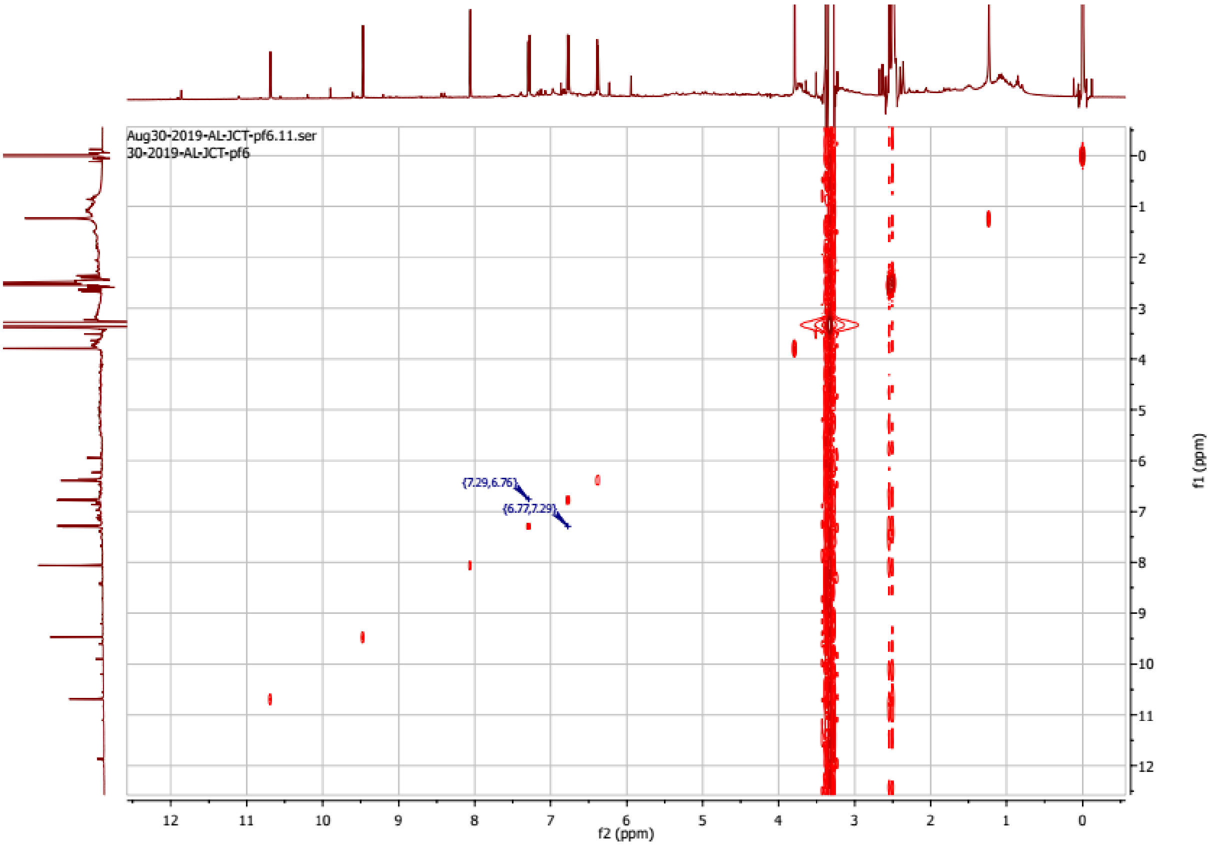

Figure S.23: Cosy spectrum (500Mhz, DMSO-D6) for compound 5

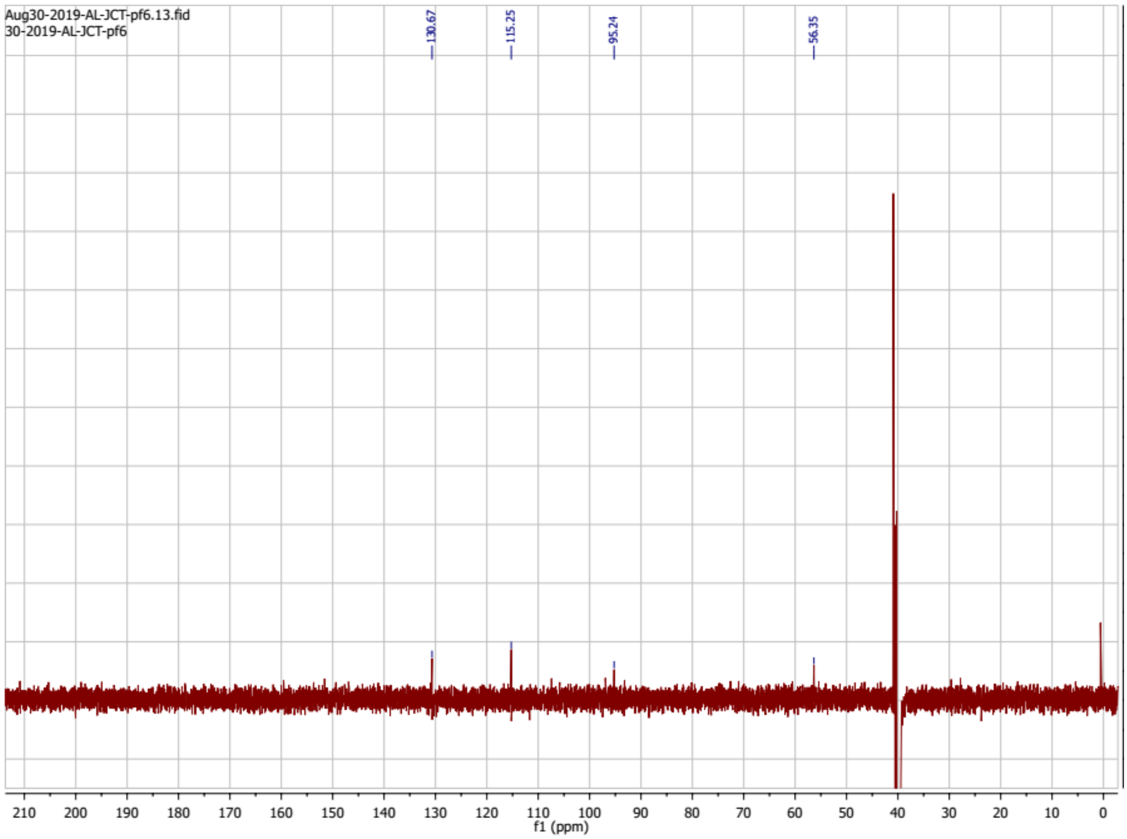

Figure S.24: 13C NMR (500Mhz, DMSO-D6) spectrum for compound 5

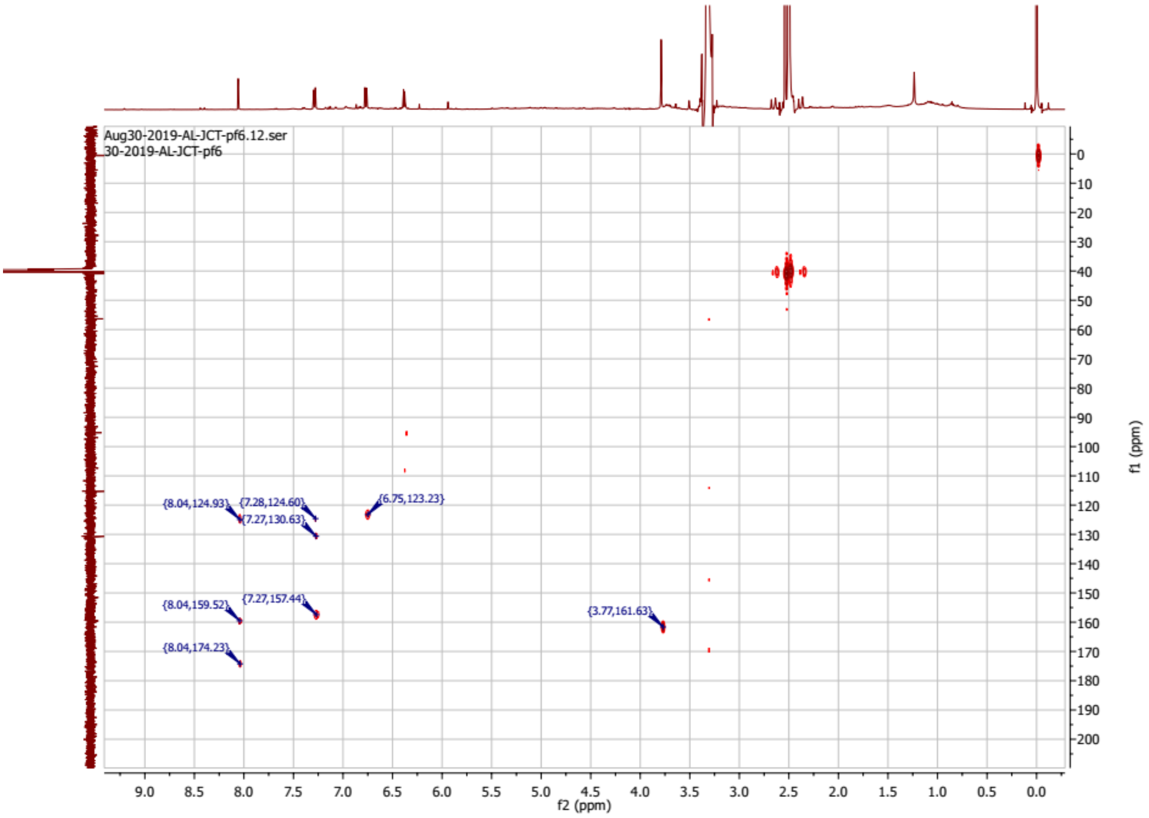

Figure S.25: HMBC spectrum (500Mhz, DMSO-D6) of compound 5

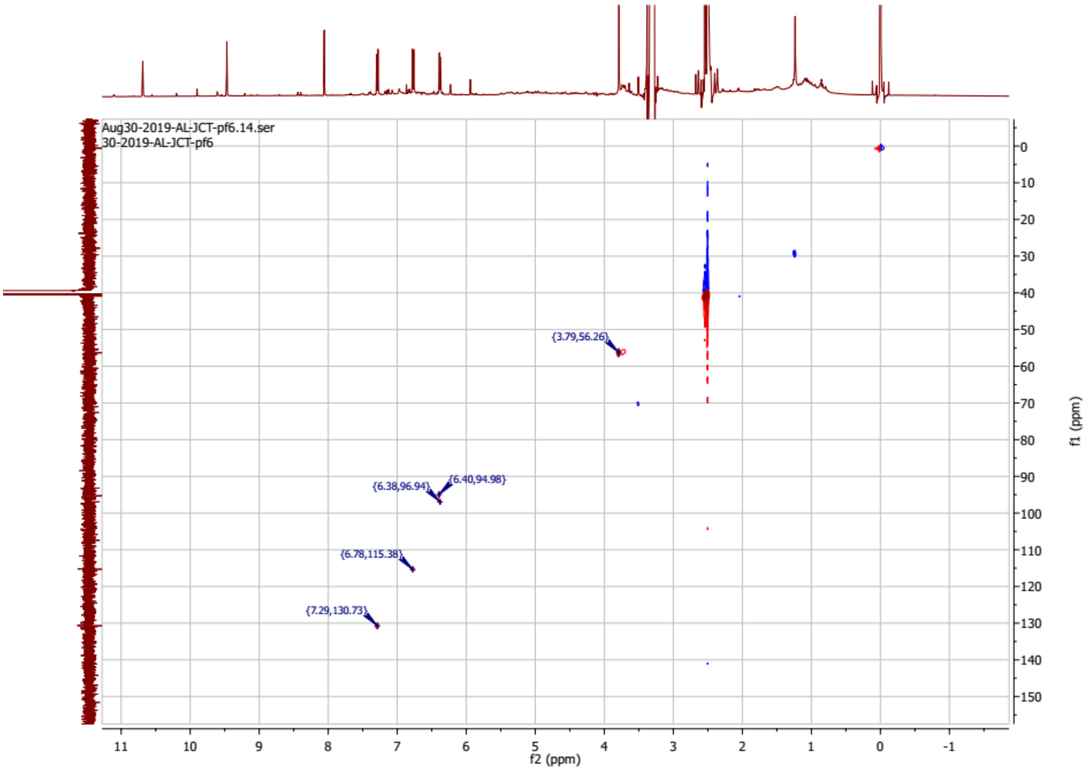

Figure S.26: HSQC spectrum (500Mhz, DMSO-D6) of compound 5

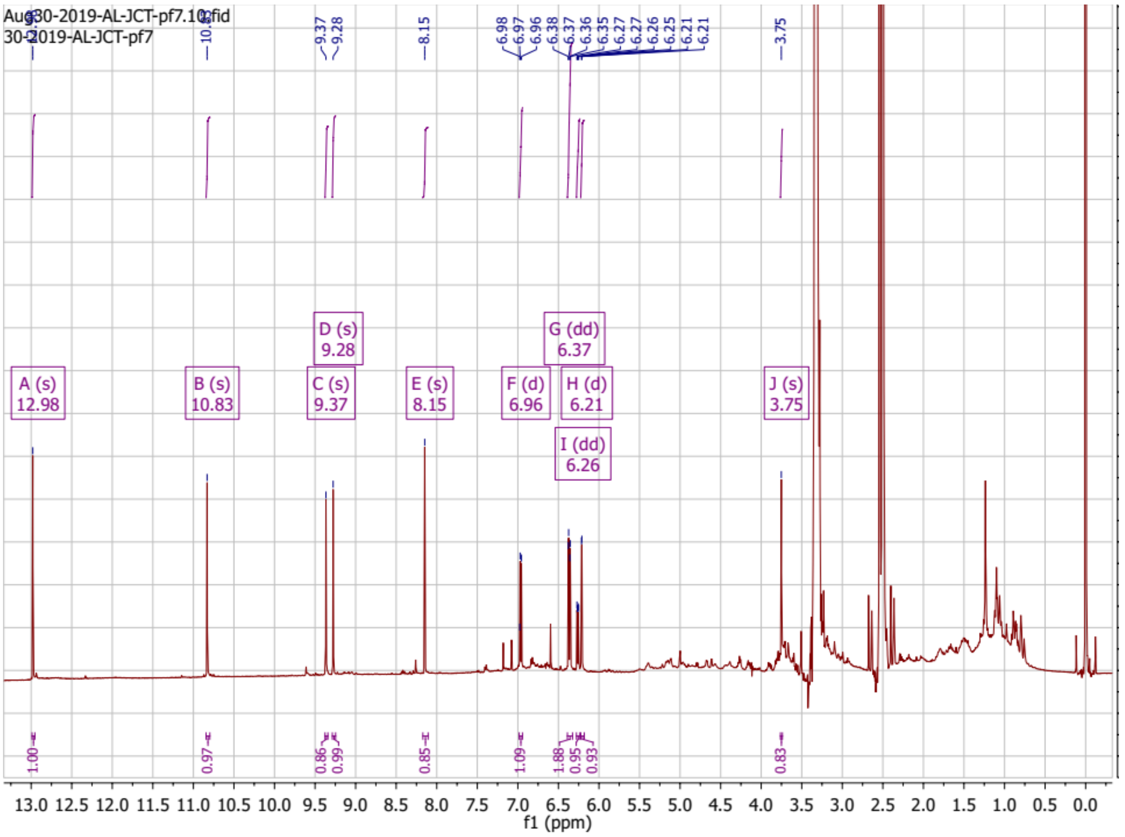

Figure S.27: 1H NMR (500Mhz, DMSO-D6) spectrum of compound 6

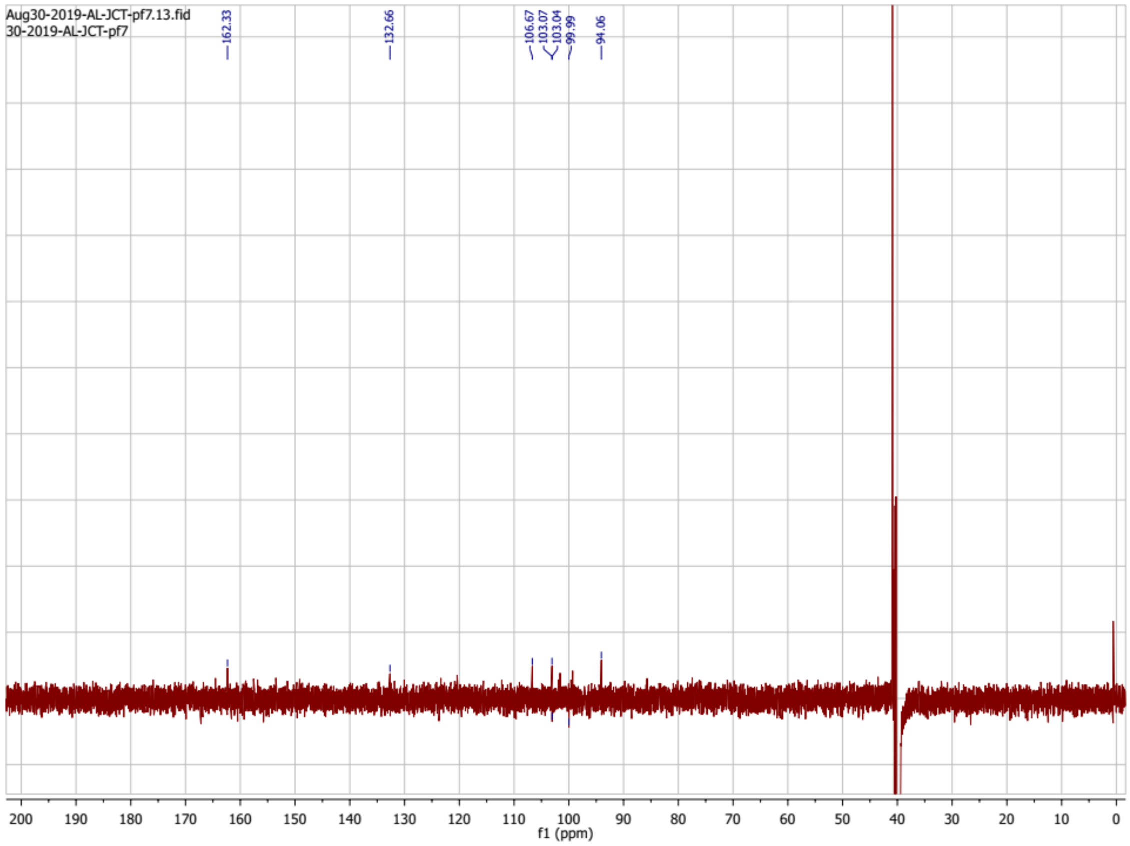

Figure S.28: 13C NMR (500Mhz, DMSO-D6) spectrum for compound 6

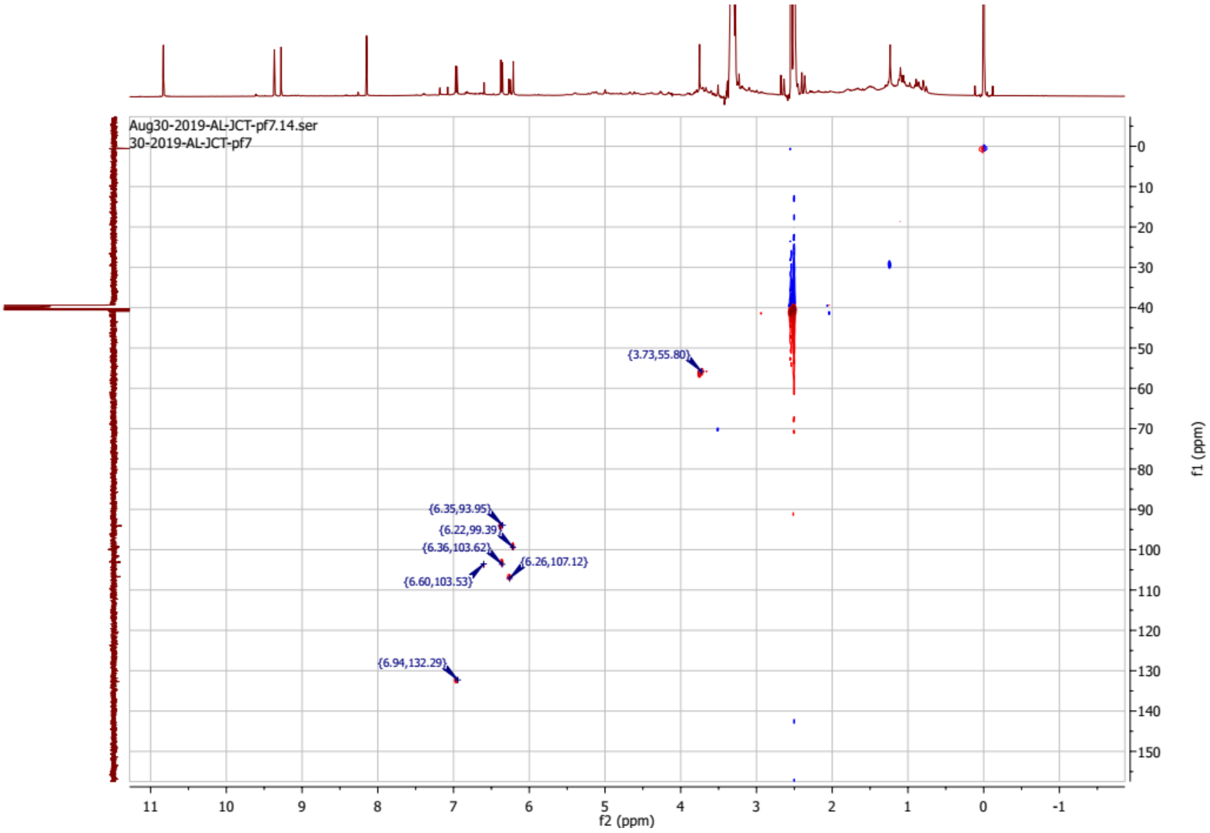

Figure S.29: HSQC spectrum (500Mhz, DMSO-D6) of compound 6

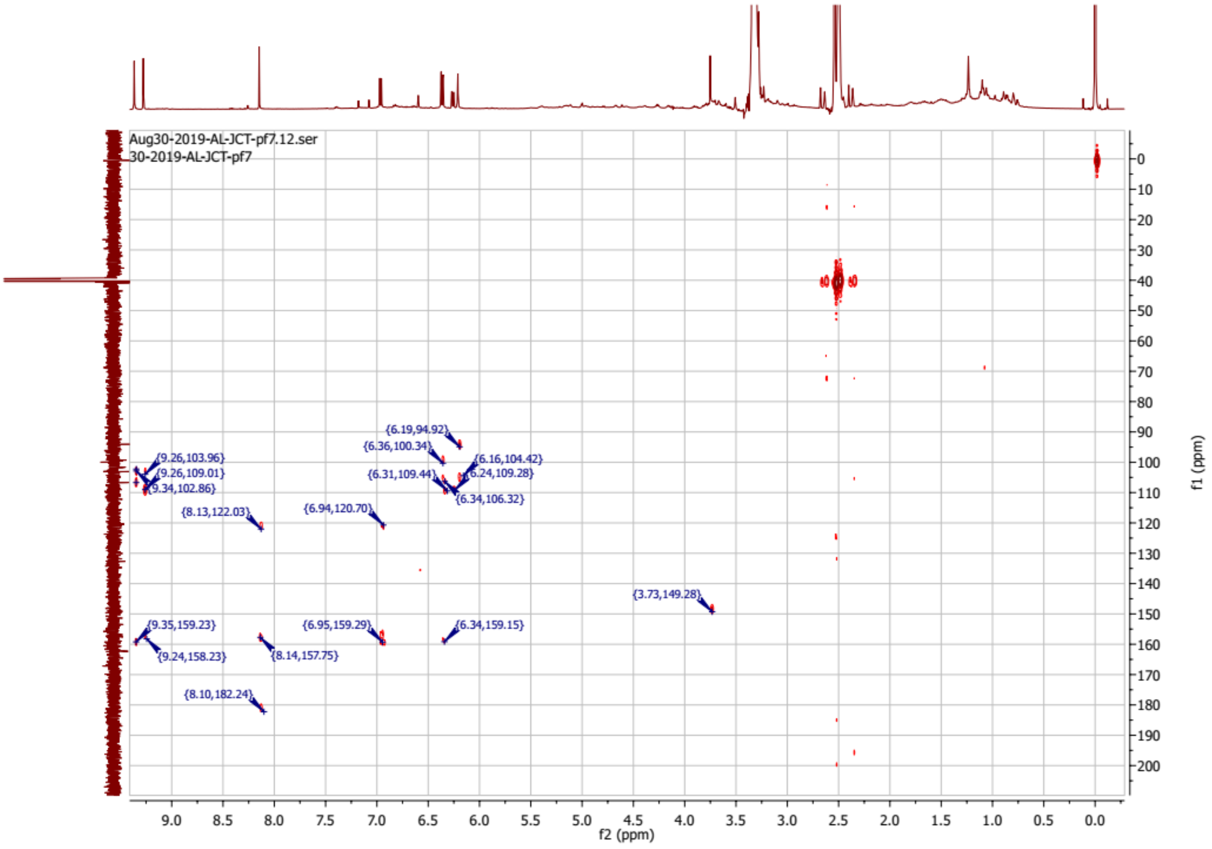

Figure S.30: HMBC spectrum (500Mhz, DMSO-D6) for compound 6

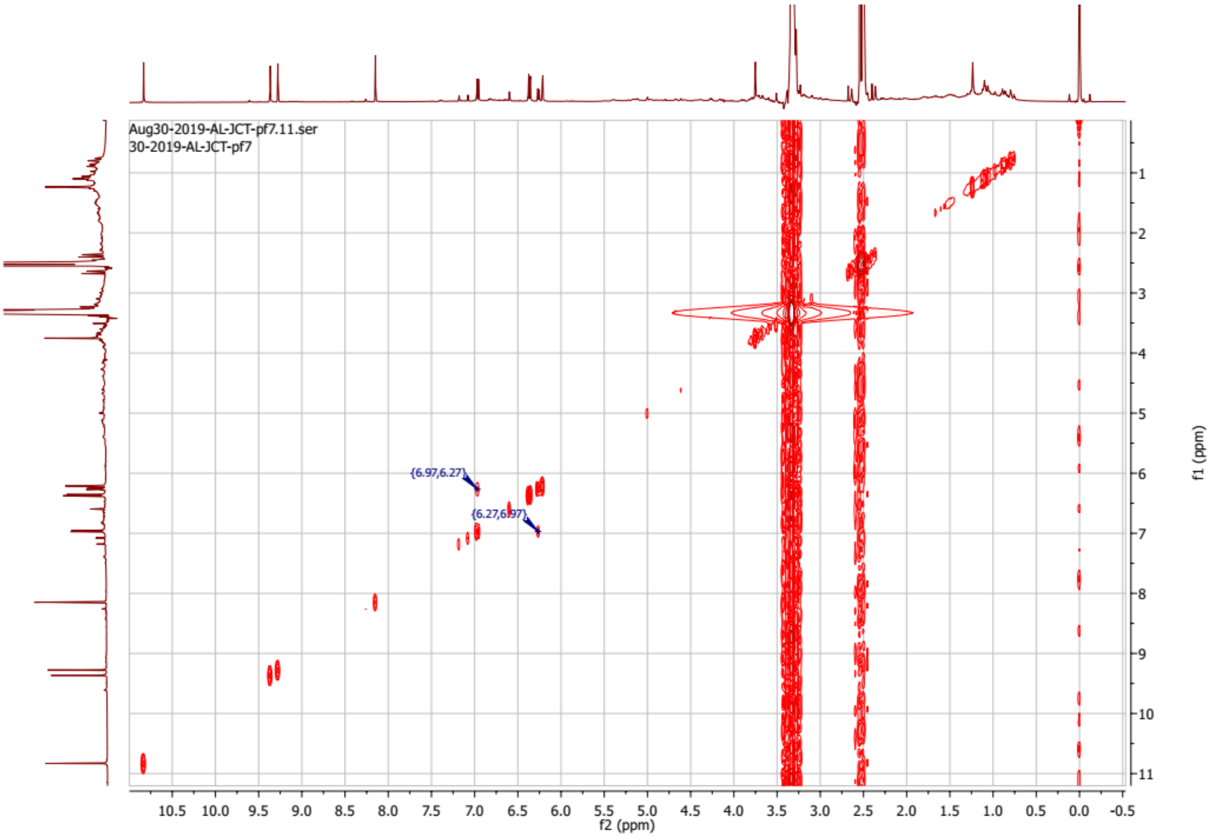

Figure S.31: Cosy spectrum (500Mhz, DMSO-D6) for compound 6

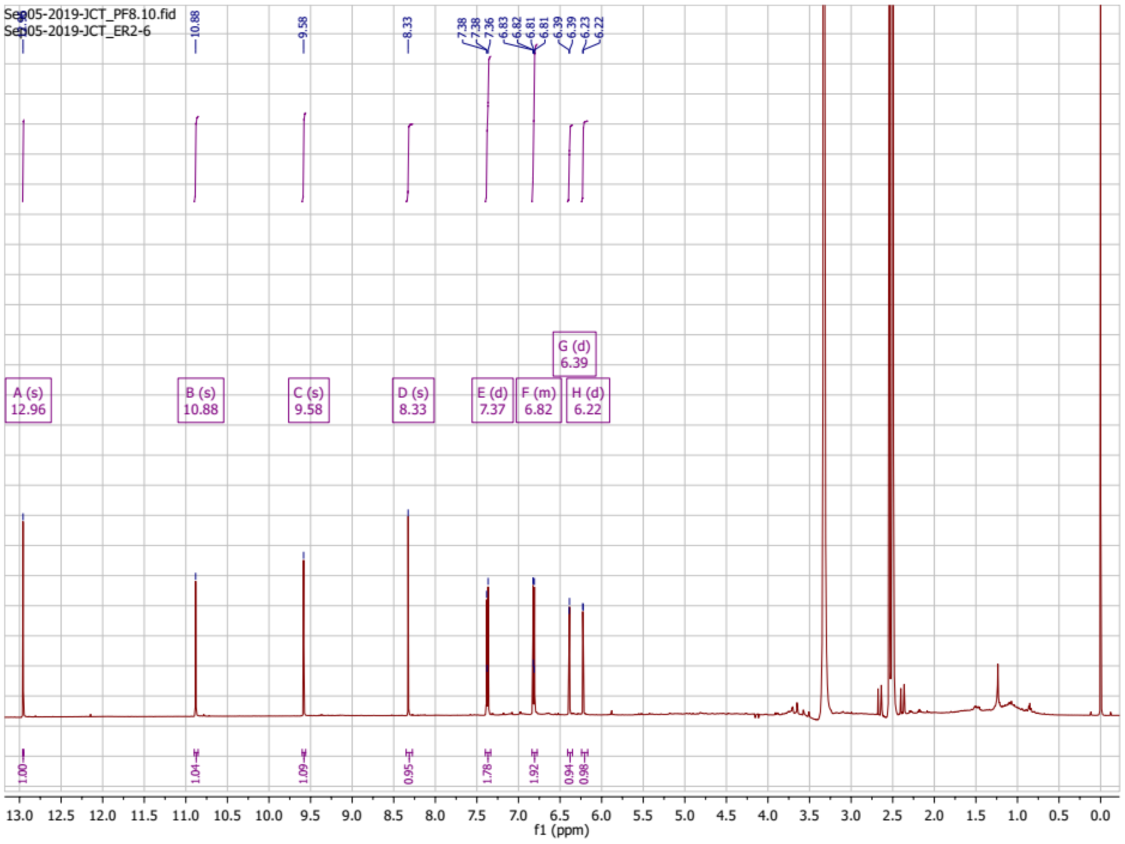

Figure S.32: 1H NMR (500Mhz, DMSO-D6) spectrum of compound 7

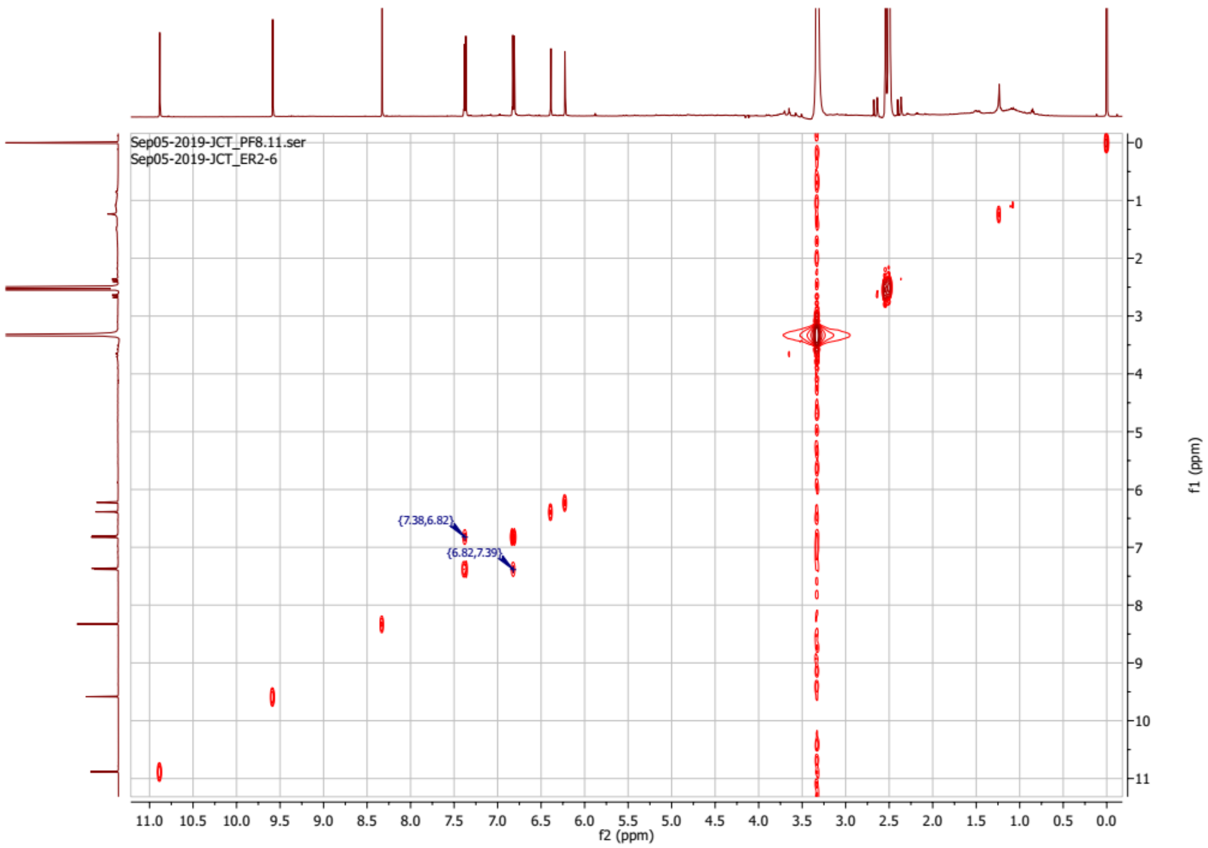

Figure S.33: Cosy spectrum (500Mhz, DMSO-D6) for compound 7

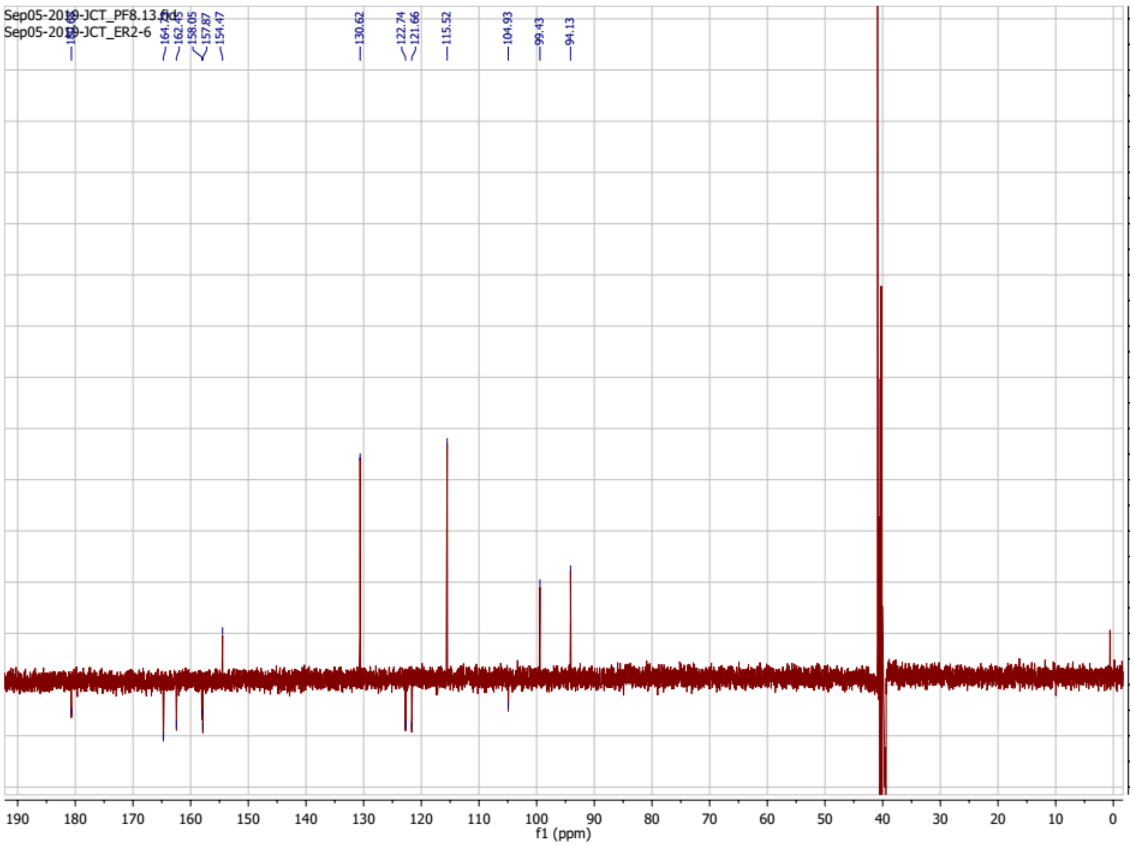

Figure S.34: 13C NMR (500Mhz, DMSO-D6) spectrum of compound 7

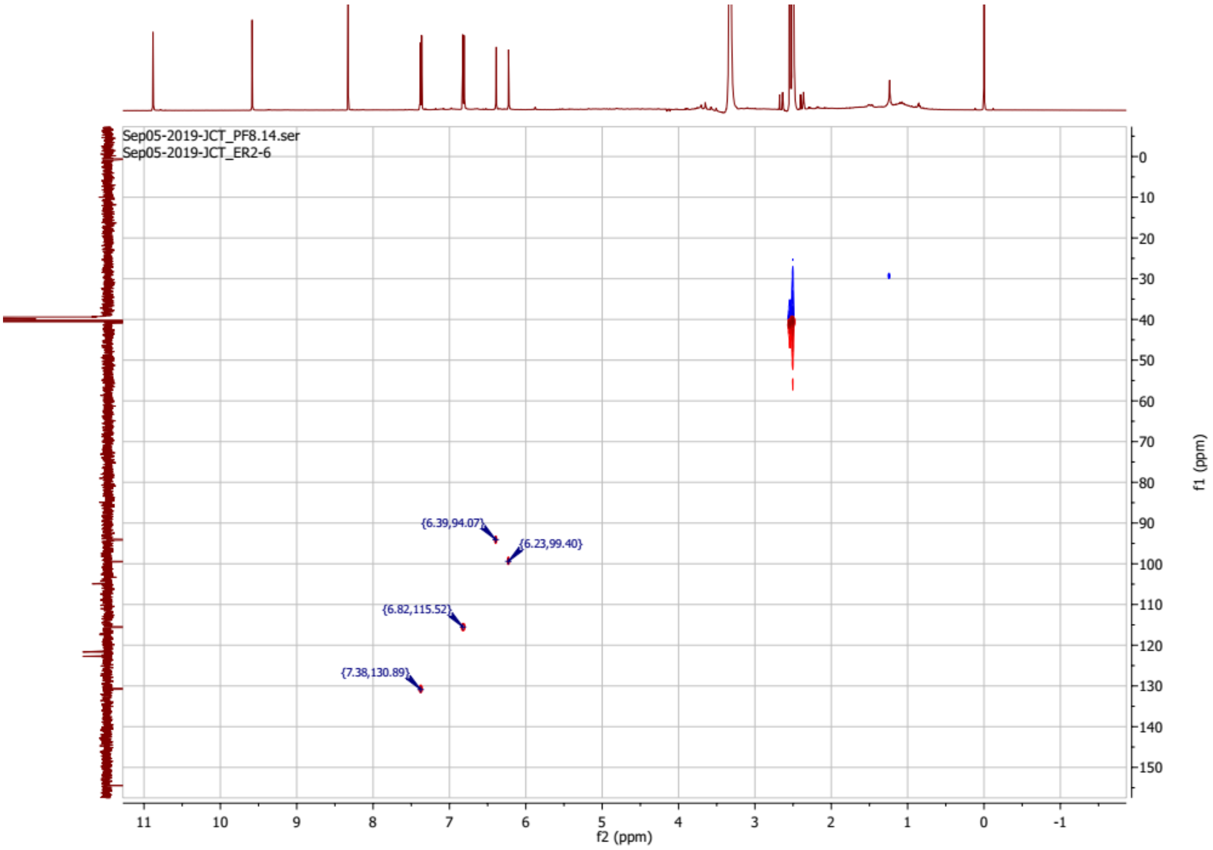

Figure S.35: HSQC spectrum (500Mhz, DMSO-D6) of compound 7

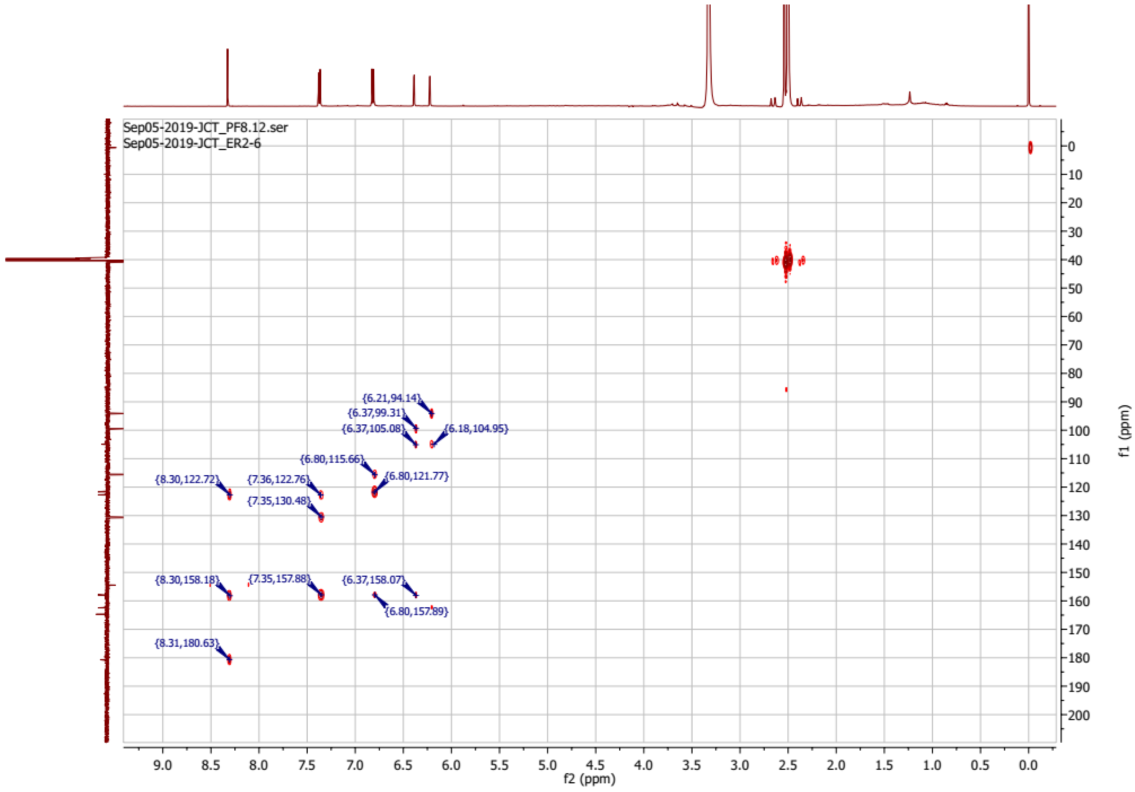

Figure S.36: HMBC spectrum (500Mhz, DMSO-D6) of compound 7
